# Evaluating R2Play, A Novel Multidomain Return-to-Play Assessment Tool for Concussion: Mixed Methods Feasibility and Face Validity Study

**DOI:** 10.2196/78486

**Published:** 2025-11-25

**Authors:** Josh Shore, Pavreet Gill, Danielle DuPlessis, Emma Kyrinis, Andrew Lovell, Kylie D Mallory, Andrea Hickling, Michael Hutchison, Sarah Munce, Kathryn J Schneider, Elaine Biddiss, Shannon E Scratch

**Affiliations:** 1 Rehabilitation Sciences Institute, Temerty Faculty of Medicine, University of Toronto, Toronto, ON, Canada; 2 Tanenbaum Institute for Science in Sport, Toronto, ON, Canada; 3 Bloorview Research Institute, Holland Bloorview Kids Rehabilitation Hospital, 150 Kilgour Road, Toronto, ON, M4G 1R8, Canada; 4 Department of Psychology, Faculty of Health, York University, Toronto, ON, Canada; 5 Faculty of Kinesiology and Physical Education, University of Toronto, Toronto, ON, Canada; 6 Department of Occupational Science and Occupational Therapy, Temerty Faculty of Medicine, University of Toronto, Toronto, ON, Canada; 7 Keenan Research Centre for Biomedical Science, St. Michael's Hospital, Toronto, ON, Canada; 8 Institute of Health Policy, Management, & Evaluation, Dalla Lana School of Public Health, University of Toronto, Toronto, ON, Canada; 9 Sport Injury Prevention Research Centre, Faculty of Kinesiology, University of Calgary, Calgary, AB, Canada; 10 Alberta Children’s Hospital Research Institute, University of Calgary, Calgary, AB, Canada; 11 Hotchkiss Brain Institute, University of Calgary, Calgary, AB, Canada; 12 Institute of Biomedical Engineering, Faculty of Applied Science and Engineering, University of Toronto, Toronto, ON, Canada; 13 Department of Pediatrics, Temerty Faculty of Medicine, University of Toronto, Toronto, ON, Canada

**Keywords:** concussion, pediatrics, return to sport, dual task, feasibility, mixed methods

## Abstract

**Background:**

Return-to-play guidelines for concussion recommend a multimodal approach to assess recovery, symptoms, exertion tolerance, and cognition. However, existing assessments do not reflect the speed or complexity of multidomain skill integration in sport. We developed *R2Play*, a dynamic multidomain return-to-play assessment tool, and previously established proof of concept by demonstrating design objectives alignment.

**Objective:**

We aim to (1) assess the feasibility of *R2Play* according to usability, reliability, practicality, and safety; (2) examine physical exertion levels during *R2Play* as a preliminary marker of face validity; and (3) understand clinician and youth perspectives on the feasibility, face validity, potential value, and challenges associated with *R2Play*.

**Methods:**

A convergent parallel mixed methods design was used. Rehabilitation clinicians were paired with youth cleared to return-to-play postconcussion to complete *R2Play* together and provide feedback through semistructured interviews. Feasibility was assessed on predefined criteria for usability (clinician ratings on System Usability Scale), practicality (assessment duration), reliability (technical issues), and safety (adverse events). Face validity was evaluated with a target of youth achieving ≥80% of age-predicted maximal heart rate or rating of perceived exertion ≥7/10. Interviews explored perspectives on feasibility and face validity, analyzed using content analysis. Quantitative and qualitative results were merged via joint display to identify areas of convergence, divergence, and complementarity.

**Results:**

Participants included 10 youth (ages 13‐20 y) with a history of concussion and 5 clinicians (n=2 physiotherapists, n=2 occupational therapists, and n=1 kinesiologist). Success criteria were met or approached for all feasibility domains. Clinician-rated usability was good-to-excellent (System Usability Scale=84.00±6.02), and youth reported that instructions were easy to learn. There were no catastrophic technical or user errors interrupting assessments. Configuration was completed in 5.74 (SD 1.09) minutes, and assessments took 26.50±6.02 minutes. There were no safety or symptom exacerbation incidents requiring assessment modification. *R2Play* elicited vigorous intensity physical exertion (peak heart rate=90.10±5.78% age-predicted maximal heart rate, peak rating of perceived exertion=5.50±1.72), with target exertion criteria met for 9/10 youth. Clinician and youth feedback confirmed that *R2Play* reflects elements of sport across physical, cognitive, and perceptual domains, making it a valuable tool for assessing readiness to return-to-play and informing rehabilitation planning for unresolved issues. Mixed methods meta-inferences provided enhanced insights regarding how to improve the usability, practicality, safety, and face validity of *R2Play*.

**Conclusions:**

Findings support the potential feasibility and face validity of *R2Play*, a multidomain assessment tool for youth with concussion, demonstrating excellent usability, vigorous physical exertion demands, and promising feedback regarding its potential to fill gaps in the return-to-play process among this initial sample from a single site. Future work is underway to establish the cross-site feasibility of *R2Play* and evaluate its content validity by establishing the physical, cognitive, and perceptual loading of assessment levels.

## Introduction

### Background

Concussion is a significant public health concern, which impacts more than 10% of Canadian school-aged children each year [1,2]. Sport is a leading cause, accounting for 30%‐40% of concussions among Canadian youth [3-5]. In Ontario, Canada, Rowan’s Law (Bill 193) guides concussion safety practices, including a mandate for medical clearance of youth returning to sport after concussion [6]. Similar policies exist in all US states [7] and are recommended across Canadian provinces [8,9]. Yet, evaluating concussion recovery and readiness for return-to-play (return-to-play) is challenging, as there is no single objective test and limited evidence-based standards to support decision-making [10-12]. Evidence suggests that current approaches are inadequate, as injury risk remains significantly elevated in the year following return-to-play postconcussion, indicating potential ongoing neurological dysfunction [13,14]. Many athletes also report feeling anxiety or fear upon return-to-play, highlighting the importance of psychological readiness [15,16]. Better tools are clearly needed to measure complete biopsychosocial recovery and guide safe return-to-play after concussion.

### Multidomain Concussion Assessment

Emerging research shows that novel assessment paradigms simultaneously targeting multiple domains of function (eg, cognitive and motor performance) can help uncover lingering subclinical neurophysiological changes after concussion [12,17]. Dual task tests involving concurrent motor (eg, walking) and cognitive (eg, backward spelling) tasks elicit subtle neuromuscular and attentional impairments among individuals with concussion that persist beyond traditional single-domain measures for several months after injury and are linked to subsequent injury risk [17-21]. Deficits include changes in gait speed or stability, cognitive accuracy, and reaction time, which are quantified in dual task costs by the magnitude of performance decrements upon introduction of a second task [17,22-24]. These performance “costs” become more pronounced as cognitive task complexity increases [25]. It is thus problematic that standard dual task tests do not reflect the speed or complexity of skill integration in sport, which involves multiplanar movement and spontaneous decision-making in response to dynamic stimuli, within an unpredictable environment [26,27]. Emerging digital approaches such as virtual reality offer promising potential for creating more realistic testing scenarios but face challenges in clinical translation (eg, cost or availability, personalization, or scoring complexity) and have yet to be adapted for return-to-play (see the review by DuPlessis et al [26] of existing multidomain assessments). Thus, ecologically relevant and clinically feasible multidomain assessments that mimic the complex demands of sport may help guide rehabilitation and safe return-to-play following concussion [26].

### *R2Play* Assessment Tool

Our team developed *R2Play,* a multidomain return-to-play assessment tool for youth with concussion, designed to help simulate the complex demands of sport within clinical settings [28]. *R2Play* was developed through a user-centered design approach involving clinicians, sport coaches, youth athletes, and families with lived experience of concussion. Through this process, *R2Play* was established as a system of low-cost tablets displaying numbers and letters that youth run between to connect an alphanumeric “trail” (1-A-2-B-3-C…) through levels with layered challenges to perception, cognition, and exertion. Heart rate (HR), perceived exertion, and concussion symptoms are monitored throughout *R2Play*, with performance measured via level completion times, errors, and multitask cost scores reflecting performance changes between levels with the addition of new challenges. Using low-cost and widely accessible technology, *R2Play* aims to provide a clinically feasible return-to-play assessment with greater ecological relevance through integration of physical skills (multiplanar exertion), cognition (complex decision-making), and psychosocial stress (performance pressure) within a dynamic, changing environment. A complete description of the *R2Play* development process and initial prototype can be found in DuPlessis et al [28]

Feasibility testing is an important step in complex intervention development that can help improve design, strengthen evaluation methods, and facilitate implementation [29,30]. For technology-based rehabilitation innovations, progressive small-scale studies involving end users are recommended to help identify and proactively address usability and feasibility issues before broader-scaled evaluation [31]. We conducted initial proof-of-concept testing for *R2Play* (version 1) with 5 clinicians and 10 youth sport participants without recent concussion history, demonstrating alignment with system design objectives, including excellent usability, moderate intensity physical exertion demands, and promising qualitative feedback regarding its potential value in clinical practice [32]. Based on this preliminary work, minor changes were made to improve *R2Play* (eg, enhancing the usability of interface screens, refining level structure, and shortening the assessment). In the present study, we sought to test the revised *R2Play* system (version 2) among youth previously cleared to return-to-play, a critical step to enable larger validation studies among youth recovering from concussion.

### Purpose and Objectives

The purpose of this study was to evaluate the feasibility and face validity of *R2Play* (version 2) among youth with concussion history and prior clearance to return-to-play. We conceptualized face validity as the extent to which *R2Play* feels like sport, a valuable early indicator of how well it reflects multidomain demands of sport and the extent to which it may apply to real-world sport environments (ie, ecological validity) [28,33]. Specific objectives were:

1. Assess the feasibility of *R2Play* in terms of usability, reliability, practicality, and safety according to the a priori success criteria described in Table 1.
2. Examine physical exertion levels during *R2Play* as a marker of face validity, with the target of youth achieving ≥80% of age-predicted maximal heart rate (HR_max_) or rating of perceived exertion (RPE) ≥7/10.
3. Understand clinician and youth perspectives on the feasibility, face validity, potential value, and challenges associated with *R2Play*.

Iterative design and testing are essential to ensure success in large-scale validation and implementation of health technologies [31]. Addressing these aims is thus intended to help optimize the design of *R2Play*, increasing the likelihood of achieving favorable outcomes in larger-scale psychometric validation, efficacy, and implementation trials.

**Table 1.** Quantitative feasibility domains and success criteria. Data sources for each domain can be found in Multimedia Appendix 1.

Domain	Definition	Success criteria
Usability	Does the *R2Play* system allow clinicians and youth to complete the assessment effectively, efficiently, and to their satisfaction?	Mean SUS^a^ score ≥80 90% of assessments completed without catastrophic user error^b^
Reliability	Does the *R2Play* system perform appropriately to administer the assessment without error?	90% of assessments completed without catastrophic technical error^b^
Implementation and practicality	Can *R2Play* be learned and administered in a timely and consistent manner?	All clinicians achieve training task completion scores >15 (75%) during a 1-hour virtual training session. Assessment configuration completed in ≤5 minutes for 90% of sessions Active assessment completed in 20‐30 minutes for 90% of sessions
Safety	Can *R2Play* be administered safely to youth being cleared for return-to-play postconcussion?	No *R2Play*-related safety incidents requiring medical attention No *R2Play* assessment sessions requiring cessation or modification due to concerning exacerbation of concussion-related symptoms

a SUS: System Usability Scale.

b See definition in Textbox 1.

Textbox 1. **Definitions and severity grading of technical errors and usability issues.**

**Event definitions**

- Technical errors: instances in which the system malfunctions or does not perform appropriately as expected (eg, unresponsive tablet, incorrect content displayed on tablet, wrong screen shown within clinician interface, and buttons within interface not functioning properly).
- Usability issues: aspects of the system or a user’s (clinician or youth) interaction with the system that make it difficult for them to independently complete an assessment task effectively and efficiently (eg, pressing the wrong button to achieve the desired goal or not understanding level instructions).

**Event severity**

- Recoverable: system or interface enters a state that the users did not intend, but they can quickly recover and continue the assessment without intervention from the research team.
- Unrecoverable: system or interface enters a state that the users did not intend, and they cannot recover without intervention from the research team. The research team resolves the issue quickly without significantly interrupting the assessment session.
- Catastrophic: assessment session is terminated and must be entirely restarted or significantly interrupted in a way that requires substantial intervention from the research team and causes a delay in continuing the assessment.

## Methods

### Design

This study followed a convergent parallel mixed methods design in which quantitative and qualitative data were collected simultaneously, analyzed separately, and merged upon interpretation with equal priority [34]. Specifically, we leveraged a descriptive observational design [35] for quantitative evaluation and used qualitative description [36] to explore participant perspectives. From a pragmatist perspective, this approach sought to obtain a comprehensive understanding of research questions through triangulation across methods and datasets [37]. It aimed to address not only whether *R2Play* could work, but also how and why it may work, with the goal of gathering data and feedback to optimize this novel assessment approach [38]. Broadly, we operationalized feasibility according to domains described by Bowen et al [39], which are reflected in both quantitative and qualitative components. The GRAMMS (Good Reporting of a Mixed Methods Study) checklist (Checklist 1) was used to guide study design and dissemination [40].

### Participants

Identical samples were used for quantitative and qualitative strands [41]. Ten youth previously cleared to return-to-play after concussions were each paired with 1 of 5 trained clinicians to complete *R2Play* together. These sample sizes reflect the exploratory nature of the quantitative objectives and align with recommendations for qualitative interview studies [42]. In our proof-of-concept work, qualitative data saturation was reached with the same sample sizes and study design [32].

Youth inclusion criteria were age 10‐25 years (consistent with WHO definition of “young person” [43]), as younger children require unique developmentally appropriate assessments [12]; experience with return-to-play process after concussion within the previous 5 years (any mechanism of injury); clinician clearance for unrestricted RTP (per current standard of practice [12]); normal or corrected to normal vision and hearing; fluent in English. Youth were excluded if they had any pre-existing conditions that could impede safe participation (eg, musculoskeletal injury or neurological or cardiovascular condition) or a developmental or learning disability that interfered with their ability to do physical activities, hear noises, follow instructions, or communicate.

Clinicians were required to have ≥1 year of experience working with youth, experience working with concussion or another acquired brain injury population, English fluency, and ability to commit to study requirements (6 h total over 4 separate days).

### Recruitment

Convenience sampling was used, in which youth were recruited internally through the Holland Bloorview Kids Rehabilitation Hospital (HBKRH) concussion services. Youth who accessed clinic services were either referred to this study by a care team member or contacted directly by the research team following discharge if they had consented to participate in the HBKRH research notification program. Additional youth were identified via snowball sampling, word of mouth, and institutional advertisements at HBKRH. Clinicians were recruited from the local community via social media, professional networks, and word of mouth. Diversity was sought in representation across disciplines and practice settings (ie, private vs public, acute vs chronic).

### Protocol

**Figure 1.**
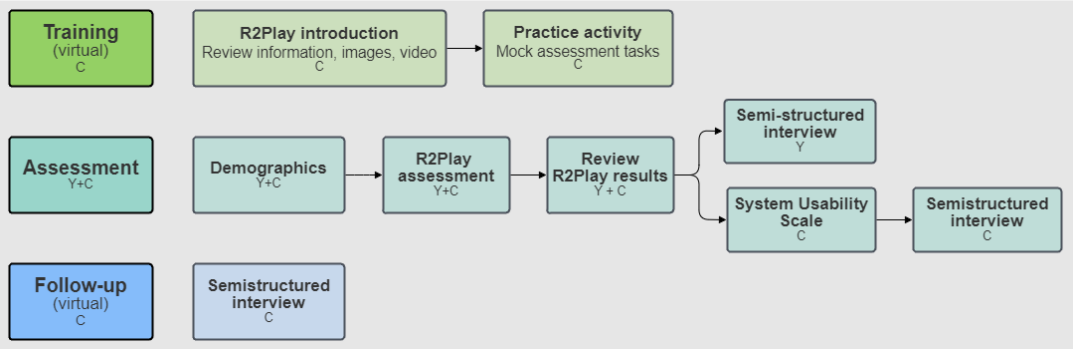
Study protocol. C: clinician participant; Y: youth participant.

Figure 1 summarizes this study’s protocol. Clinicians first attended a 1-hour virtual training session to learn to administer *R2Play*. Training was led by the primary author (JS) and involved a brief presentation, demonstration of the system interface, and practicing key tasks in facilitating *R2Play* (Checklist 2). Following training, clinicians were paired with a youth participant to complete *R2Play* together in person at HBKRH. After finishing the assessment, the clinician and youth participants reviewed results together with a research team member and then completed separate semistructured interviews to provide feedback. To enable practice and reflection, clinicians were asked to complete 2 assessment sessions with different youth. Within 1 week after their second session, clinicians completed an in-depth follow-up interview (30‐60 min) to reflect further on their experience and perspective, considering both *R2Play* sessions.

### Data Collection Measures

#### Demographics

Youth completed a brief demographic form collecting their age, sex, gender, sport participation (weekly hours), number of diagnosed concussions, and time to return-to-play. Clinicians were asked to report their age, gender, discipline, years of experience, practice setting, and client age range.

#### Clinician Training Task Activity

A standardized checklist (Checklist 2) was used by the training facilitator (JS) to record completion of 10 key tasks during the training practice activity. Task completion was scored as completed with ease (2), completed with help (1), or not completed (0) [28]. Total completion score was calculated by summing individual task scores (maximum score of 20).

#### *R2Play* Assessment

##### Protocol

**Figure 2.**
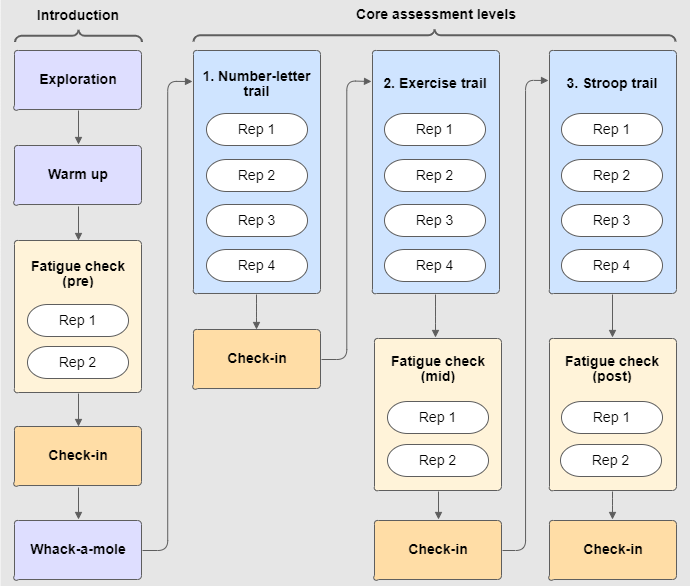
*R2Play* (version 2) assessment protocol. The introductory phase helps orient participants to the *R2Play* system and learn the assessment task. The core assessment levels make up the backbone of assessment scoring. Rep: repetition.

**Figure 3.**
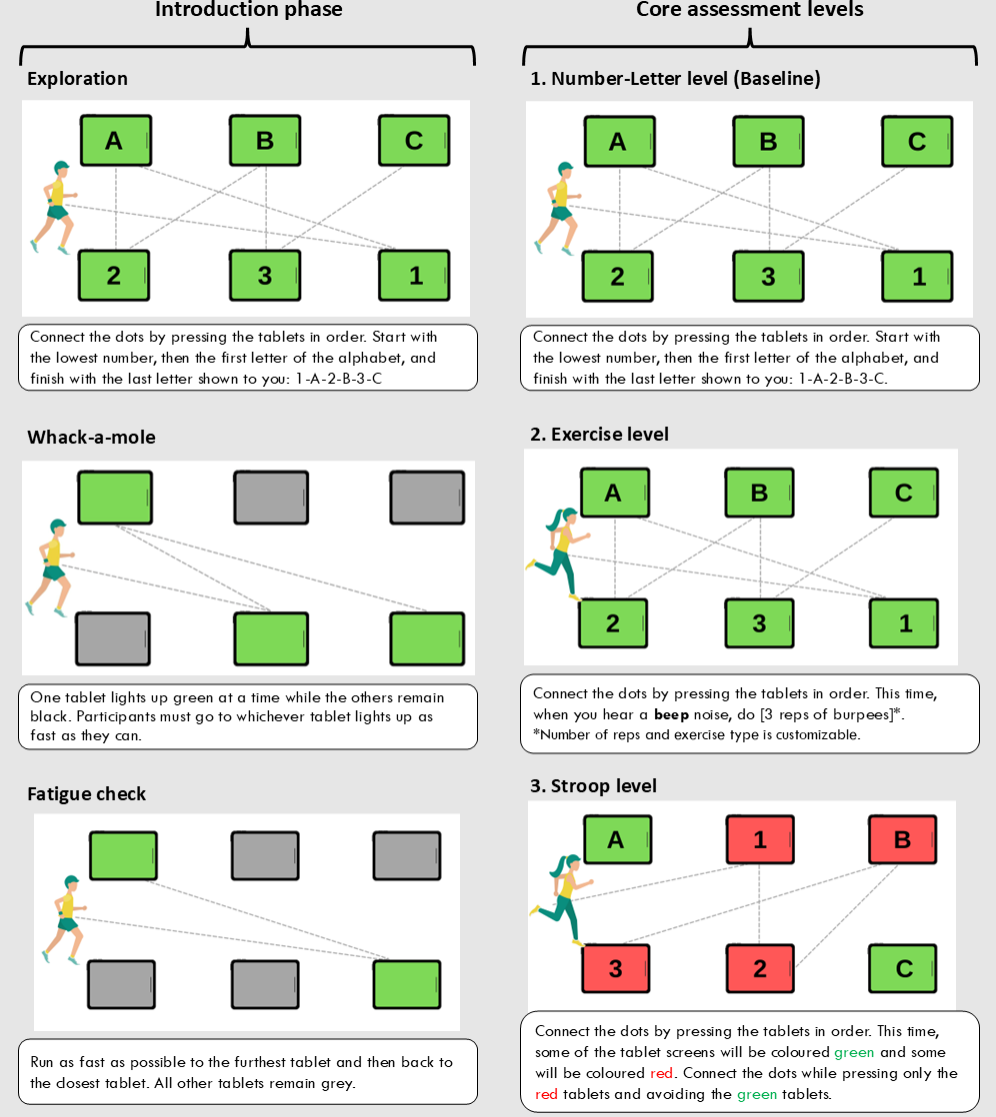
*R2Play* (version 2) assessment level descriptions. For the core assessment levels, 1 repetition comprised 2 cycles of the same 6-character sequence (1-A-2-B-3-C), for a total of 12 tablet selections. Trail sequences were programmed to have running distance between correct tablet taps standardized to 45 m (±1%) for all number-letter and exercise level repetitions and 30 m (±1%) for all Stroop level repetitions (due to green tablet omissions). Across all levels, the distance traveled per correct tablet selection was approximately 7.5 m. Rep: repetition.

**Figure 4.**
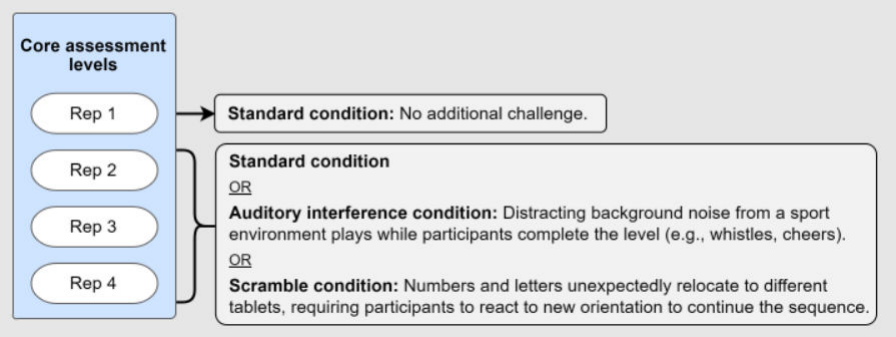
Repetition conditions for the core *R2Play* assessment levels. Within each level, the first repetition is the standard condition. Repetitions 2‐4 are a randomized order of another standard condition, the auditory inference condition, and the scramble condition. Rep: repetition.

An overview of the *R2Play* assessment (version 2) can be found in Figure 2. Briefly, the assessment consisted of an introduction phase with 3 activities to orient participants to the *R2Play* system and task, followed by 3 core assessment levels (number-letter, exertion, and Stroop) with varied physical and cognitive demand complexity. Between these core levels, there were 3 fatigue checks to capture any changes in sprint completion time. A description of each assessment activity can be found in Figure 3. Each core level consisted of 4 repetitions: 2 standard condition repetitions and 2 repetitions that introduced layered perceptual challenge conditions (Figure 4) [44]. Level instructions were explained to youth using 4 training methods within the *R2Play* interface: (1) verbal instructions; (2) introducing trail sounds; (3) video demonstration of the level, and (4) tracing activity to practice the number-letter pattern. Brief check-ins were conducted after each level, in which participants rated their perceived exertion and changes in concussion symptoms (see measures below). HR was also monitored throughout the entire assessment and streamed live in the clinician interface.

##### Scoring

The *R2Play* assessment is scored via level completion times, errors (ie, incorrect tablet selections), and HR. Completion time is calculated in seconds and measured relative to the number of tablet selections in the level (ie, seconds/tablet). Based on these metrics, multidomain cost scores are calculated that reflect performance changes between levels and conditions as new challenges are added. Cost scores are expressed as the percentage change in score between a baseline and more challenging level or condition (calculations detailed in Multimedia Appendix 2).

### Heart Rate

Youth wore a Polar H10 chest strap monitor (Polar Electro) to capture their HR throughout *R2Play*. Average and peak HR (beats per minute) were calculated for each participant and level.

### Rating of Perceived Exertion

Youth reported their RPE during check-ins immediately after each *R2Play* level using the 10-point children’s OMNI scale, a validated tool for measuring RPE among exercising youth [44].

### Concussion-Related Symptom Evaluations

#### Post-Concussion Symptom Inventory

Before and after *R2Play*, youth completed the 26-item adolescent Post-Concussion Symptom Inventory (PCSI) [45] to assess the presence and severity of concussion-related symptoms across physical, cognitive, emotional, and sleep or fatigue domains. Symptoms were rated on a 6-point Likert scale from “not a problem” (0) to “severe problem” (6). Overall PCSI symptom score was calculated by summing all rated symptoms (maximum of 156).

#### Symptom Check-In

After each *R2Play* level, youth completed a brief check-in to assess symptom changes, with the following response options: no symptoms (0), some symptoms but did not get worse (1), symptoms got worse (2), symptoms so much worse I had to stop (3).

### System Usability Scale

Immediately after finishing each *R2Play* assessment, clinicians completed the System Usability Scale (SUS), a validated measure of subjective usability [46]. The SUS contains 10 items rated on a 5-point Likert scale ranging from strongly disagree (1) to strongly agree (5). Item responses are each assigned a score contribution from 0‐4, which are summed and multiplied by 2.5 to produce a total SUS score of 100. A total SUS score of 68 is considered average, 68‐80 is good, and ≥80 excellent [47].

### Assessment Session Recordings

Screen recordings of the *R2Play* clinician software interface and video recordings of the entire assessment sessions were captured to provide insight regarding participant interactions with the system and to enable further investigation of technical or usability issues.

### Field Notes

A structured template was used by research team members during all *R2Play* assessment sessions to record field notes capturing any technical errors, usability issues, or support required by participants.

### Semistructured Interviews

Interviews explored participants’ experiences with *R2Play* and perspectives on its feasibility, face validity, potential value, and challenges. Youth were asked to reflect on the acceptability of *R2Play*, usability, demand (ie, perceived value), practicality (eg, ability to learn rules), and face validity (ie, whether *R2Play* felt like playing sports). Clinician postsession interviews focused on initial reactions to *R2Play* in terms of usability, training requirements, and perceived value. Clinician follow-up interviews were structured around multiple domains of feasibility (acceptability, usability, demand, implementation, practicality, adaptability, expansion, and safety) [39] and face validity. Interview guides can be found in Multimedia Appendix 3. All interviews were audio recorded, transcribed verbatim, and checked for accuracy.

### Analysis

#### Quantitative

Feasibility (objective 1) was evaluated based on predefined criteria for usability, reliability, practicality, and safety (Table 1). These domains were chosen from Bowen et al [39] and other literature [31,48] to address the most pertinent uncertainties at this stage, with the success criteria set based on proof-of-concept results where possible [32,49,50]. Assessment duration and exertion data were ascertained from automated system logs and analyzed using Microsoft Excel (Microsoft Corp) and R statistical software (R Foundation). Quantitative feasibility data were summarized using descriptive statistics and 95% CIs where appropriate.

Observational analysis of video and screen recordings was performed using a standardized data extraction sheet to identify technical and usability issues (Multimedia Appendix 4). Two authors (JS and AL) independently reviewed recording footage from 2 *R2Play* sessions and met to compare findings and resolve discrepancies. Each researcher then reviewed 4 more session recordings independently. Once all sessions were reviewed, the researchers met again to discuss and confirm identified events, collaboratively resolving any uncertainties. Field notes were referenced to provide additional context about identified events. Finally, both researchers worked together to group similar events and tally their occurrences. Identified issues were discussed with the project engineer to determine causes and implement solutions.

Physical exertion (objective 2) was evaluated with the target of youth achieving a vigorous intensity level at any point during the assessment (≥80% HR_max_ or RPE ≥7) as a marker of face validity [49]. These thresholds align with clinical exercise testing guidelines and exertion testing protocols for concussion [50,51]. Exertion data were plotted and visually inspected to identify trends across assessment levels. There was no missing data across all quantitative analyses.

#### Qualitative

Interview transcripts exploring participant perspectives (objective 3) were analyzed separately for youth and clinicians via qualitative content analysis using the feasibility and face validity domains as a framework for analysis [52]. A total of 2 transcripts were collaboratively coded by 2 authors for clinician postsession and follow-up interviews (JS and EK), and youth interviews (JS and DD). Codes were initially derived inductively from the data, without applying any theoretical framework and using the participants’ own words wherever possible. Preliminary clinician and youth codebooks were then transferred to NVivo qualitative analysis software (Lumivero) and flexibly applied to the remaining transcripts by the lead author (JS), allowing for the addition of new codes to capture unique concepts. Once all transcripts were coded, reviewers organized the codes into categories that specifically addressed the feasibility and face validity domains. Strategies to enhance rigor included collaboratively coding initial transcripts, verifying emergent codes with another reviewer, discussing categories within the research team, and retaining an audit trail of analysis [53,54].

#### Mixed Methods Integration

Quantitative and qualitative strands were integrated at the levels of design, methods, reporting, and interpretation [34,55]. Within this convergent design, strands were initially linked by identical sampling of the same participants (ie, connecting) [41,55]. Strand-specific data for feasibility and face validity were analyzed separately, with results reported narratively using a weaving approach [55]. Strands were merged via joint display to identify areas of convergence, divergence, and complementarity [55,56]. Data was triangulated across sources (eg, SUS, video and screen recordings, interviews, and field notes) and samples (ie, clinicians and youth) to aid interpretation and enhance credibility [57]. Multimedia Appendix 3 provides a joint display of integrated data highlighting the specific quantitative and qualitative data addressing study aims. We used the decision tree from Younas and Durante [58] and guidance from Aschbrenner et al [59] to select appropriate joint displays based on our design, mixing purpose, and integration techniques. Joint displays were constructed according to essential elements recommended by Guetterman et al [56]

### Ethical Considerations

Ethics approval was obtained from HBKRH (REB #20‐099) and the University of Toronto (REB #40694). All participants provided voluntary informed written consent. Efforts were undertaken to maintain privacy and confidentiality of participant data and identities. Participants received compensation in the form of gift cards.

## Results

### Overview

Five clinicians and 10 youth (ages 13‐20 y) with concussion history participated (Tables2  3). Quantitative and qualitative results for each feasibility domain and face validity are presented within the subsections below, each followed by a brief description of key findings from mixed methods integration (ie, meta-inferences) presented via joint displays.

**Table 2.** Clinician participant characteristics.

Clinicians (C)	Age range (years)	Sex	Clinical discipline	Practice setting	Experience (years)	Days between study sessions
C1	30‐35	F	Physiotherapy	Private	5‐10	16
C2	25‐30	F	Kinesiology	Public and private	3‐5	4
C3	25‐30	F	Occupational therapy	Private	3‐5	3
C4	25‐30	F	Physiotherapy	Public and private	0‐3	71
C5	30‐35	F	Occupational therapy	Private	5‐10	28

**Table 3.** Youth participant characteristics.

Youth (Y)	Age (years)	Sex	Gender	Sports played (hours per week)	Number of consussions reported	Time since last concussion (months)	Approximate time to return-to-play (days) for latest concussion
Y1	14	M^a^	Boy or man	Hockey, soccer, and volleyball (19 total)	6	2	Unknown
Y2	16	F^b^	Girl or woman	Softball (5-6), ultimate frisbee (4), running (1-2), and swimming (2-3)	1	20	30
Y3	19	F	Girl or woman	Snowboarding (0‐10) and Muay Thai (4-12)	2	59	120
Y4	16	F	Girl or woman	Basketball and volleyball (6 total)	2	36	30
Y5	14	M	Boy or man	Hockey (6), baseball (3), and snowboarding (8)	2	26	14
Y6	16	F	Girl or woman	Hockey (4-5) and gymnastics (2)	3	54	120
Y7	13	F	Girl or woman	Soccer (6), hockey (6), and badminton (10)	5	6	150
Y8	15	M	Boy or man	Hockey (4), snowboarding (6), swimming, golf, and tennis	1	50	21
Y9	13	F	Girl or woman	Volleyball (4)	1	2	7
Y10	20	F	Girl or woman	Soccer (8)	2	60	60

a M: male.

b F: female.

### Feasibility

#### Usability

##### SUS

Mean total SUS score across all sessions was 83.75 (SD 4.45; 95% CI 80.99‐86.51), surpassing the success target of 80. Usability ratings remained relatively stable from clinicians’ first (SUS=83.50±2.85; 95% CI 81.00‐86.00) to second sessions (SUS=84.00±6.02; 95% CI 78.72‐89.28). An SUS score >80 places *R2Play* within the 90‐95 percentile range of usability, corresponding to “good-to-excellent” usability [60]. Across both sessions, items rated least favorably were “I felt very confident using the system” (mean 3.90, SD 0.74) and “I needed to learn a lot of things before I could get going with this system” (mean 2.50, SD 0.97; reverse weighting).

##### Usability Issues From Observational Analysis

Usability issues identified in the video and screen recording analysis are shown in Table 4. Overall, fewer usability issues were documented for clinicians’ second assessment sessions (n=16) compared to their first (n=23), and a higher proportion of issues were recoverable in their second sessions (53% vs 17%). No usability issues met the definition of catastrophic (Textbox 1).

**Table 4.** Usability issues identified from observational analysis of video and screen recordings.

Severity^a^		Frequency	Description
First assessment session (n=23; 17% recoverable)			
Catastrophic		0	N/A^b^
Unrecoverable (n=19)^c^			
		8	The researcher intervened to provide direction because the clinician did not deliver the appropriate level’s instructions
		4	The clinician needed help from the researcher to navigate the software interface
		4	The youth required an explanation of the instructions from the researcher
		2	The clinician asked for clarification from the researcher about the level’s instructions
		1	The youth false-started (moved before the start cue)
Recoverable (n=4)^c^			
		3	The youth required further explanation of instructions from the clinician while completing a level
		1	The clinician had difficulty navigating the software interface but was able to recover independently
Second assessment session (n=15; 53% recoverable)			
Catastrophic		0	N/A^b^
Unrecoverable (n=7)^c^			
		2	The clinician needed help from a researcher to navigate the software interface
		2	The clinician asked for clarification from the researcher about the level’s instructions
		1	The youth false-started (moved before the start cue)
		1	The clinician did not provide appropriate level instructions
		1	The clinician asked for clarification about check-in response options
Recoverable (n=8)^c^			
		3	The clinician had difficulty navigating the software interface but was able to recover independently
		3	The youth required further explanation of instructions from the clinician while completing a level
		2	The tablet needed to be readjusted

a Severity definitions can be found in Textbox 1. Briefly, unrecoverable errors required intervention from the research team, while recoverable errors could be resolved by the clinician or the youth participant themselves.

b N/A: not applicable.

c Subtotal.

##### Qualitative Interview Findings (Clinicians)

###### Usability Overview

Clinicians found *R2Play* highly usable, describing the interface as “well designed,” “intuitive,” and “easy to use.” They noted it became easier with practice, and they felt more comfortable in their second session despite the time between sessions (3‐71 d). Confidence in using *R2Play* was primarily limited by a lack of familiarity with the assessment protocol and level instructions.

###### Usable Aspects

On-screen prompts and automation made it particularly easy for clinicians to navigate the interface: “I think the way it is set up… kind of just the next thing is automatic, I think makes sense from a usability standpoint” (C5). They also appreciated that all components of the assessment were “embedded” within the interface (eg, PCSI) so they did not need to manage additional applications or supplies. Other usable aspects included the multimodal level training screens, time-stamped observation notes, and “cheat sheet” handout resources.

###### Usability Challenges

Clinicians found that following the overall flow of the *R2Play* assessment protocol and delivering level instructions was most challenging. For example, one clinician said, “It took me a little while to get used to the protocol. I’d get confused as to what I was getting the athlete to do sometimes… just getting your head wrapped around the flow and how to direct the athlete and work through the different stages” (C1). Some clinicians also reported difficulty navigating results screens and explaining the cost scores to youth.

###### Usability Suggestions

To support consistent delivery of level instructions, a standardized script embedded within the interface was recommended. Areas for increased automation, prompts, and guidance within the interface were also highlighted (eg, level training screens). Knowledge mobilization tools such as lay summaries, visuals, and interpretation guidelines were recommended to help communicate cost score results to youth and families.

### Qualitative Interview Findings (Youth)

#### Rules and Instructions

Most youth found the instructions easy to learn and follow. Some reported initial confusion about aspects of the task, for example, the need to complete 2 cycles of the 6-character sequence, but they gained familiarity through repetition of tasks across assessment levels. Video and audio demonstrations were seen as particularly helpful for learning instructions. For earlier stages of concussion recovery, more visuals and further simplification of instructions were recommended.

#### Tablet Button System

Youth described the tablet buttons as easy to use, despite some concerns regarding their stability and sensitivity. For example, before becoming familiar with the system, youth reported initial apprehension about tapping the tablets without toppling them, or being unsure how much force was needed to register a tablet selection. One participant noted that her long fingernails were a potential usability issue as they made it difficult to select tablets while running.

### Reliability

#### Technical Errors

A total of 7 technical errors were recorded and managed by the research team without data loss. There was 1 catastrophic error in which multiple tablets lost connection to the system mid-assessment, significantly interrupting active assessment time (Table 5). One unrecoverable error occurred in which the HR monitor disconnected, requiring researcher intervention. Recoverable errors included unregistered tablet selections (n=3) and buttons within the clinician interface not functioning properly (n=2).

**Table 5.** Duration of configuration and active assessment for *R2Play* sessions. Configuration time includes assessment initiation, customization, and baseline measures collection. Clinicians were not required to perform system set-up in this study (ie, arranging tablets or connecting the system). Active assessment is the time during which a participant is completing *R2Play* levels.

Session	Configuration, (minutes)	Active assessment, (minutes)	Total, (minutes)
First assessment session			
C1	12.75^a^	32.19^a^^b^	44.94
C2	4.89	27.03	31.92
C3	5.07	31.43^a^	36.50
C4	3.80	21.41	25.22
C5	7.90^a^	30.44	38.34
Mean (SD; 95% CI)	6.88**^a^** (3.61; 3.71‐10.05)	28.50 (4.43; 24.62‐32.38)	35.38 (7.36; 28.93‐41.83)
Second assessment session			
C1	6.94^a^	27.03	32.97
C2	4.29	24.96	29.24
C3	5.02	30.95	35.97
C4	6.99^a^	27.84	34.84
C5	6.44^a^	21.75	28.19
Mean (SD; 95% CI)	5.74 (1.09; 4.78‐6.69)^a^	26.50 (3.42; 23.50‐29.51)	32.24 (3.41; 29.25‐35.23)
All sessions			
Mean (SD; 95% CI)	6.31 (2.59; 4.04‐8.58)^a^	27.94 (4.06; 24.38‐31.50)	34.40 (5.66; 29.29‐39.21)

a Target feasibility success criteria were not met (Table 1).

b Active assessment interrupted by technical error (loss of system connection).

#### Qualitative Interview Findings (Clinicians and Youth)

Overall, *R2Play* was seen to run smoothly with minimal technical issues. Technical errors observed by clinicians and youth included unregistered or delayed tablet selections and unresponsive tablets. Clinicians also identified minor issues within the interface, including training videos and audio clips not functioning properly.

### Implementation and Practicality

#### Clinician Training Task Completion

All clinicians achieved a training task completion score ≥18/20 (90%) and were able to fully or partially complete all training tasks. The most common task in which all clinician participants required assistance was “train a new client on a given level of the *R2Play* assessment” (n=5 received partial points). While they were all able to deliver basic level instructions, points were deducted due to missed details (eg, not explaining repetition conditions), or ineffective use of multimodal training methods (video demo, trace-it-out, and trail sounds). The second most common task requiring help was “report and explain a specified cost score from an *R2Play* assessment to a client” (n=2 received partial points).

#### Assessment Duration

Table 5 presents the duration of assessment configuration and active assessment [1] Overall, the target of 20‐30 minutes of active assessment time was met for 8/10 (80%) sessions, while the target of ≤5 minutes of configuration time was only met for 5/10 (50%) sessions. Total *R2Play* duration was slightly lower for clinicians’ second sessions (mean 32.24, SD 3.41 min) compared to their first (mean 35.38, SD 7.36 min).

#### Qualitative Interview Findings (Clinicians)

##### Time

Clinicians spoke about the need to balance the desire for prolonged exertion against the practicality of assessment length. They appreciated measuring performance over a longer interval, in contrast to existing tests: “Sometimes our tests are only like 10 minutes, and they could clear it... So, I think the fact that it’s a bit longer of an assessment and you’re measuring over time is really helpful” (C3). However, the time required for set-up, configuration, assessment, and results review was seen as a potential barrier to clinical implementation. An hour-long appointment was thought to be required for *R2Play*, which was seen as a challenge in some practice structures, though some clinicians felt that the comprehensive nature of *R2Play* made it appropriate as a stand-alone final return-to-play clearance discharge assessment.

##### Space and Set-Up

Physical space was described as a barrier to conducting functional assessments such as *R2Play* in clinical environments. Setting up *R2Play* was seen as a potential challenge due to the equipment components and standardized positioning, which could amplify time constraints. Quick and easy set-up was thus important to clinicians, who also recommended designated assessment spaces and support staff to assist with set-up. Rehabilitation hospitals, university clinics, and specialty programs were seen as appropriate settings with suitable space and staffing to support *R2Play*.

##### Cost

Some clinicians thought the cost of *R2Play* could be prohibitive for individual providers and small organizations. They described a need to demonstrate the value of *R2Play* relative to existing tools to justify its cost and suggested discounted bundle packages for purchasing equipment, software, and training to improve affordability.

### Safety

#### Adverse Events

While no adverse events occurred during *R2Play* sessions, potential safety concerns were noted in four sessions, including participants slipping on the floor while running (n=3) or lunging beyond the tablet trail and narrowly avoiding collision with other equipment (n=2).

#### Concussion-Related Symptoms

Median PCSI symptom score decreased from 10.5 (IQR 5‐14, range 5‐17) to 6 (IQR 5‐12, range 1‐29) from pre- to post-*R2Play* assessment. Most (9/10) participants reported either “no symptoms” or “some symptoms but did not get worse” for all postlevel symptom check-ins, indicating no change in symptoms throughout *R2Play*. One participant (Y1) reported “symptoms got worse” (mild headache) but was able to continue. No sessions were stopped or modified due to worsening symptoms. Full PCSI and symptom check-in data can be found in Multimedia Appendix 5.

#### Qualitative Interview Findings (Clinicians)

##### Overview

Clinicians thought *R2Play* would be very safe if used with the right individuals, at an appropriate stage of recovery. They provided 3 broad categories of recommendations to ensure safety.

##### Preparticipation Screening and Warm-Up

Some clinicians recommended prerequisite criteria or assessments that youth should clear before attempting *R2Play*. This included minimal symptom burden, aerobic exercise tolerance, and appropriate vestibular function (eg, vestibular or ocular-motor screening). For example, 1 clinician discussed how *R2Play* could build on prior exercise testing:

Something like a Buffalo concussion [treadmill] test would be good to do [before *R2Play*]… it would be helpful if you knew they could exert themselves to a certain point and have minimal symptoms, let’s layer in complexity now with *R2Play* and that might make it safer

[C3]

Given the physical demands of *R2Play*, musculoskeletal screening and thorough physical warm-ups were also recommended.

##### Monitoring Tolerance

Clinicians emphasized the importance of frequent check-ins during *R2Play* to monitor changes in symptoms and HR. One clinician suggested standardized stopping criteria: “If there are certain criteria, like if their symptoms jump this much, if their HR jumped this much, if there are things in there that more uniformly a clinician would be stopping it, I don’t think it’s unsafe” (C3). Adequate rest breaks and opportunities to pause or stop testing were also seen as important to ensure comfort for youth who may be deconditioned or symptomatic postconcussion.

##### Controlled Testing Environment

Clinicians affirmed the need to consider the testing environment to reduce risk for injury. Specifically, ensuring an open space without tripping hazards, an appropriate floor surface to prevent slips, and appropriate footwear.

### Qualitative Interview Findings (Youth)

Youth reported feeling very safe while completing *R2Play*. They appreciated frequent check-ins and breaks, providing opportunities for rest and the option to stop if needed, which were recognized as important for youth recovering from a concussion. Some youth initially felt nervous due to the novelty or complexity of *R2Play*, which gradually subsided after the first few tasks, underscoring the importance of the introduction phase before core assessment levels. As youth became more comfortable, they also felt their performance improved: “I was anxious when coming in, but I think after the first set of stuff, I was pretty comfortable just because it was easy to grasp and follow … I felt like there was a very notable link between my anxiety going down and performance going up” (Y6). Three youth mentioned slipping and sliding on the floor as a potential safety risk or performance impediment.

### Acceptability

#### Qualitative Interview Findings (Clinicians)

##### Comprehensive Assessment

Clinicians described *R2Play* as a comprehensive assessment because it simultaneously addresses multiple factors in being ready for return-to-play after concussion and involves integration across domains of function. They appreciated how it provides a practical method to create more dynamic testing environments that better reflect the demands of sport:

It brings together all the necessary elements that we need to look at to make a really good, informed decision for readiness. So, what it does differently is it has all the input from different domains of function and things that will influence performance, which is something that isn't included in current testing and standardized protocols.

[C1]

##### Enjoyable and Engaging

Clinicians felt that *R2Play* would be enjoyable for most youth athletes who are not symptomatic. Specific aspects that they highlighted as making it particularly enjoyable for youth were its interactivity, gamification, challenge, competitiveness, and introduction of new challenges without becoming repetitive. Nevertheless, clinicians suggested that enjoyment could vary, as youth who lack confidence or are not psychologically ready for return-to-play may be hesitant to engage in *R2Play* due to fear of symptoms or reinjury. One clinician discussed how *R2Play* could help build rapport with clients through interactive activity:

I honestly think it’s helpful to build rapport with your client… there’s lots of opportunities to cheer them on or provide reassurance or encouragement… compared to like the Buffalo Concussion Test, which I think is very helpful, but I either chat with them while they're on the treadmill, but then they're also trying to focus, and then I'm just asking them questions every minute… so in terms of client rapport, there’s not a lot of time or aspect for that. So, I thought this one did that, which was fun.

[C3]

##### Customizability

Clinicians appreciated built-in options for customizing the *R2Play* assessment, such as the number of tablet selections in the trail and adapting the system layout to different clinical spaces. Some expressed a desire for more customizability related to the height of tablet targets and the ability to skip, repeat, or combine levels based on areas most relevant for each client:

One thing that could be helpful… something a bit more customizable to layer in certain features. So, if you really wanted to challenge somebody, or if they're playing an even higher level of sport, and just having the option to like, put the auditory on the whole time *and* do the Stroop *and* do the scramble… just to have more options, I guess, or more freedom to use your clinical judgment.

[C3]

### Qualitative Interview Findings (Youth)

#### Comprehensive and Realistic

Youth described *R2Play* as a comprehensive test of readiness for return-to-play that was “more realistic to going back to sports” (Y1). They found it a good reflection of elements required for return-to-play, with over two-thirds of youth specifically mentioning combined physical and cognitive demands as an aspect they especially liked about it. For example, “I like that it’s exercise, but you also have to think at the same time, because that’s definitely a big part of [RTP]. I think that was good to put those two things together.” (Y3).

#### Enjoyable and Engaging

Youth described *R2Play* as more enjoyable than other assessments in their recovery. They appreciated the “active,” “engaging,” and “interactive” nature of *R2Play,* which they framed in contrast to more passive traditional assessments such as walking, treadmill tests, and questionnaires: “[This was] definitely a lot more active. When I was doing [my recovery], I think it was a lot of just walking, like I never got actually out of breath… this one was a lot more fun, a lot more interactive and engaging.” (Y8). The gamification of *R2Play* and involvement of technology (ie, tablets) were seen as particularly appropriate for young people. Task variety and the introduction of new challenges throughout *R2Play* were also highlighted as elements that increased engagement and enjoyment.

### Demand

#### Qualitative Interview Findings (Clinicians)

##### Limitations With Existing Approaches to Return-to-Play

Clinician interviews highlighted gaps in current approaches to the return-to-play process, including a lack of standardized criteria for determining readiness and limitations with existing assessments. They described a lack of consistency in RTP clearance, whereby organizations or providers devise their own protocols, leading to subjective decision-making largely based on intuition. Elements of readiness to RTP that clinicians felt were not sufficiently addressed by existing assessments included multiplanar movement, multidomain integration (ie, thinking while exercising), and sensory sensitivity (eg, noise). They emphasized that these components are not considered until athletes progress to sport-specific training in which the clinician is typically not present:

The change of direction, acceleration, deceleration, turning movements, quick pivots, those things are where you'll start to see challenge, certainly in a sport situation, and aren't usually incorporated until the athlete is on the field or with their team in an environment that you're not able to evaluate other than just asking them how they did.

[C1]

##### Assessing Sport Tolerance

Clinicians discussed how *R2Play* could help assess clients’ tolerance to sport-like activity within a controlled setting before returning to unpredictable sport environments. For this purpose, clinicians would consider the athlete’s response to dynamic exertion and any symptom changes throughout *R2Play* as an indicator of readiness for return-to-play:

If [a person’s] symptoms are exacerbating when they're doing this task, that’s a pretty good indicator that maybe they're not ready to be doing something at that high intensity or that length of time.

[C5]

##### Using Cost Scores to Assess Multidomain Skill Integration

Clinicians appreciated the cost score approach to assessing multidomain skill integration and emphasized the importance of interpreting them within the context of the individual’s overall condition and their own clinical judgment. They also saw value in observing youths’ movement during testing to help contextualize cost score results in relation to the performance requirements of their specific sport or activity. Further research was emphasized to support clinical use of cost scores, including understanding psychometric properties (validity and reliability), creating normative datasets, establishing clinically significant cutoffs, and developing knowledge mobilization tools to support interpretation.

##### Results Can Inform Tailored Return-to-Play Guidance

Based on cost score results and potential symptom exacerbation during testing, clinicians felt that *R2Play* could help identify individual areas of weakness and inform personalized guidance to address any unresolved consequences of injury. For example, “If I’m noticing that when there is auditory noise or there is a scramble, it’s a huge discrepancy, then they’re probably not ready to go into an environment where they have to make a lot of cognitive decisions on a quick basis. So maybe they’re not ready for passing drills that involve someone else coming from a different direction, or they’re not ready to go into a busy gym yet.” (C4).

### Qualitative Interview Findings (Youth)

#### Limitations With Existing Approaches to Return-to-Play

Similar to clinicians, the youth discussed limitations with existing approaches to the return-to-play process based on their experiences. Though the confidence they reported at the time of RTP varied, most youth expressed concerns about the tests and exercises they did during their recovery. Many described the process as focusing on either physical or cognitive aspects, without adequate attention to both domains and their integration. One youth discussed how testing physical-cognitive integration could have helped in their recovery:

I wish something like this had been around when I was trying to return to sport because things like reaction time and all that stuff and my ability to think while moving around was not something that was really ever assessed… it would have made the process a lot smoother.

[Y6]

#### Value of *R2Play* for Assessing Return-to-Play Readiness

Youth discussed the value of *R2Play* for assessing tolerance to sport-like exertion. This included monitoring symptoms, HR, or any “slow-down” in their performance to enable clearer return-to-play decisions and determine appropriate exertion levels based on their progress. Some youth felt this opportunity to test their abilities within a controlled setting could have improved confidence in RTP: “I think [doing *R2Play*] would have decreased a lot of my anxiety going back and a lot of the uncertainty of like, how am I going to react? I had no clue.” (Y6).

#### Timing of *R2Play* Assessment

Given its complexity and demands, youth felt that *R2Play* would not be appropriate for early stages of recovery. Rather, they envisioned it toward the end of recovery as a final step before returning to full sport participation:

I feel like [*R2Play*] could be used during the end… when the patient is almost 100%, they could do the testing just to see if there’s any setbacks with exercise or anything

[Y5]

### Integrated Mixed Methods Feasibility Findings

To address our intended mixing purposes of comparing (triangulation across datasets), expanding (elucidating broader understandings), and enhancing (increase meaningfulness of interpretations)*,* we constructed a side-by-side comparison joint display (Table 6) to illustrate integrated mixed methods findings (meta-inferences) within the feasibility domains of interest [58,59].

**Table 6.** Side-side comparison joint display of mixed methods integration findings for feasibility domains.

Number	Domain	Quantitative evidence^a^	Qualitative evidence^a^	Meta-Inference^a^
1	Usability	SUS:^b^ mean SUS score was 83.75 (SD 4.45), within the 90‐95 percentile range of usability and corresponding to a grade of “A” for “good-to-excellent” usability. Usability issues: there were no catastrophic usability issues and few (n=7) unrecoverable usability issues in clinicians’ second assessment session.	Usability overview (C^c^): clinicians found *R2Play* highly usable and described it as “well designed,” “intuitive,” and “easy to use.” Tablet button system (Y^d^): Youth found the tablet buttons easy to use. “I think kids [are] a lot more used to using tablets… I didn’t have any trouble [using the system].” [C2] Rules and instructions (Y): most youth reported the rules of *R2Play* were easy to learn and follow. “It was easy [to learn the rules] … it’s pretty simple, like you figure it out… it’s pretty easy to pick up.” [Y1]	Concordance: *R2Play* is highly usable for both clinicians and youth. The interface is easy for clinicians to navigate, and youth can easily understand the instructions and complete required tasks using the tablet button system.
2	Usability	SUS: items rated least favorably were “I felt very confident using the system” (mean 3.90, SD 0.74) and “I needed to learn a lot of things before I could get going with the system” (mean 2.50, SD 0.97)	Usability challenges (C): clinician confidence was limited by a lack of familiarity with the *R2Play *protocol and instructions. “I don’t think it was anything difficult. I think it was more, just familiarity… just not intuitively knowing what comes next.” (C5)	Expansion: lack of familiarity with the *R2Play *protocol, rather than navigating the interface, reduced clinician confidence and perceived usability.
3	Usability and practicality	Usability issues: there were fewer usability issues in clinicians’ second assessment session (n=16) compared to their first (n=23), and a greater proportion of issues were recoverable (53% vs 17%). Assessment duration: total *R2Play *duration was lower for clinicians’ second sessions (mean 32.24, SD 3.41 min) compared to their first (mean 35.38, SD 7.36 min).	Easy to use and learn (C): *R2Play *was easier, and clinicians felt more confident in their second session. “I feel like my familiarity with the system was a lot better this time... it was much more seamless.” [C4] With more familiarity in their second session, clinicians were better able to guide participants through *R2Play*. “The more familiar you are with it, the faster you’re going to get it up and running and the faster you’ll be able to let the patient know and understand what they’re doing.” [C1]	Expansion: *R2Play *becomes easier to use and faster to administer with practice due to greater familiarity with navigating the interface and delivering level instructions.
4	Usability and practicality	Training task activity: training tasks in which clinicians needed the most help were (1) Train a client on a level of the *R2Play* assessment (n=5); and (2) Report and explain a specified cost score from an *R2Play* assessment to a client (n=2).	Usability challenges (C): aspects of *R2Play *that were challenging for clinicians included explaining level instructions and assessment results. “The main [challenge] was delivering the tests exactly and just knowing what kind of things to emphasize so the participant was able to understand what we’re asking.” [C2] “I still kind of fumbled with the results screens, the cost scores and stuff, I think that’s where I was still kind of second-guessing.” [C3]	Concordance: clinicians need support to follow the overall *R2Play* protocol, deliver instructions in a standardized manner, and interpret and explain cost score results in a way that is understandable and meaningful to youth and families.
5	Practicality	Assessment duration: the target of 20‐30 minutes active assessment time was met for 8/10 (80%) sessions, including all clinicians’ second sessions.	Time (C): the time required for set-up, configuration, assessment, and results review was seen by clinicians as a potential barrier to clinical implementation of *R2Play*. *“*I think one of the biggest challenges will be the length of it… for it to seamlessly integrate into current practice, I think it might be long” [C1]	Discordance: target duration was met for most sessions, though clinicians had concerns about the length of *R2Play*, suggesting a need to reconsider the targets and further explore practicality.
6	Safety	Adverse events: there were no adverse events recorded across all sessions. Concussion symptoms**:** total PCSI symptom score decreased from pre- to post-*R2Play*, and no *R2Play *sessions were stopped or modified due to worsening symptoms.	Safety (C): clinicians thought *R2Play* would be very safe if used with the right individuals at an appropriate stage of recovery. “It’s very safe, you are in a controlled environment, you have that heart rate monitor on them, you are checking on their symptoms… if there are any concerns, you’re in that controlled environment to manage that.” (C2) Safety (Y): youth reported feeling very safe while completing *R2Play*. “I felt very comfortable and very safe.” [Y10]	Concordance: *R2Play *appears safe with appropriate precautions in place. Among youth previously cleared to return-to-play after concussion, *R2Play *does not appear to cause symptom exacerbation.
7	Safety	Adverse events: near misses were recorded for (1) participant slipping while running (n=3) and (2) participant lunging beyond the tablet trail and narrowly avoiding other equipment (n=2).	Controlled testing environment (C): clinicians highlighted the testing environment as important to limit hazards for slips and trips. “The only thing I noticed from the last one was just the slippery floors, so that’s something [to be] mindful of.” [C2] Safety (Y). 3 youth mentioned slipping as a potential safety risk.	Concordance: testing environment is an important safety consideration for *R2Play,* including floor surface and other potential hazards.

a Text before the colon indicates quantitative data source (column 3), qualitative category or subcategory (column 4), or meta-inference labeling (column 5).

b SUS: System Usability Scale.

c C: clinician.

d Y: youth.

### Face Validity

#### Physical Exertion

**Figure 5.**
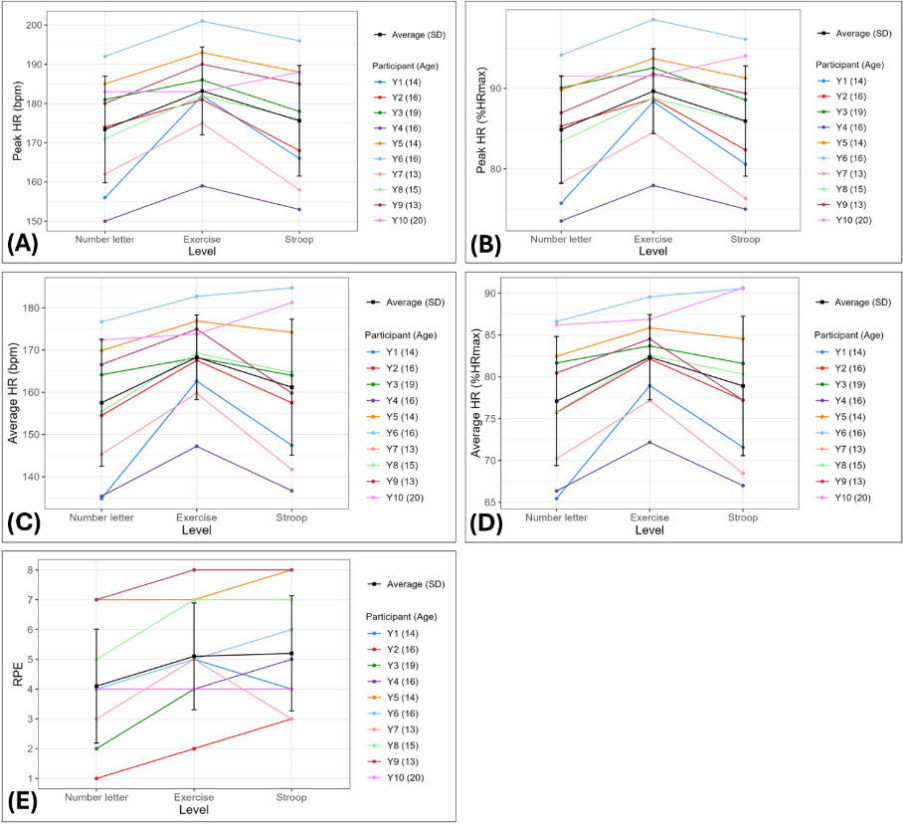
HR and RPE for each participant across core *R2Play* assessment levels. HR is expressed as the peak raw HR measurement (A), peak percentage of age-predicted maximal HR (B), average HR (C), and average percentage of age-predicted maximal HR (D) across all repetitions within each level. RPE (E) was captured retrospectively during breaks between levels. HR: heart rate; %HR_max_: percentage of age-predicted maximal heart rate; RPE: rating of perceived exertion; Y: youth participant.

Figure 5 presents HR and RPE data for each participant across *R2Play* levels. All participants achieved vigorous intensity physical exertion levels (peak HR=78%‐99% HR_max_, panel B). Target exertion criteria (≥80% HR_max_ or RPE ≥7) were met for 9/10 participants. As expected, HR was generally highest in the exercise level, while RPE was elevated in the exercise and Stroop levels compared to the baseline number letter level.

#### Qualitative Interview Findings (Clinicians)

##### Represented Elements of Sport

###### Overview

Clinician feedback regarding elements of sport represented in *R2Play* is presented with sample quotes in Table 7 and summarized in the subsections below.

**Table 7.** Clinician feedback regarding elements of sport represented in *R2Play*.

Domain category and subcategory	Sample quote
Physical	
Maximal exertion	“Getting that really fast acceleration… where they’re basically running full tilt, is helpful because a lot of times when you’re putting people through a return-to-play, you’re getting them on a bike and telling them to cycle at a certain amount, they’re not hitting that 80%‐90% of heart rate max, and sometimes it’s at those higher levels that you start to see some symptoms.” [C4]
Sustained exertion	“The thing I liked about [*R2Play*] was that it was long enough that you have to be *working*, exerting your body for a longer period of time, which is more indicative of sport… sometimes our tests are only like 10 minutes long and they could clear it… So, I think the fact that it’s a bit longer of an assessment and you’re measuring over time is really helpful.” [C3]
Multiplanar movement	“It’s really the only one that I’m familiar with that allows as much functional movement… more of the sport-specific movements that you’d be expecting them to have to be capable of… like change of direction, acceleration, deceleration, a lot of the turning movements, the quick pivots” [C1]
Quick movements	“You were just also assessing like the ability to click the [tablets] at a certain speed. So, I kind of just made note like if they were able to make those fast movements.” [C2]
Cognitive	
Reaction time	“I do think it’s good at getting… like quick, reactionary kind of stuff which I think is sometimes missing with return-to-play assessments.” [C3]
Divided attention	“You’re seeing like, how well they can tolerate like a sudden change in the environment and just getting distracted and then coming back to it.” [C2]
Spontaneous decision-making	“It forces the participant to be reactive rather than just be able to kind of go through like an obstacle course or something that they know exactly what’s going to happen.” [C4]
Multitasking	“I think the multitasking part is quite effective… It’s the first one that I’ve done that has the multitasking element.” [C4]
Perceptual	
Visual stimulation	“And then the visual stimuli. If you are, let’s say at a hockey game, and there’s the bright lights… any arena where there are lights or changes, you can see that [from *R2Play*] and you can make note of like, what kind of triggers are there.” [C2]
Background noise	“…those background noises… a lot of athletes, when they do return to play, the audio is like a major thing as well, especially someone with concussion with noise sensitivity.” [C2]

###### Physical Domain

Clinicians emphasized high exertional load, multiplanar movement, and quick motor responses as physical aspects of sport reflected in *R2Play*. They appreciated how it elicited maximal and sustained exertion, which were seen as important aspects of sport missing in current protocols. Dynamic multiplanar movement was also seen as unique compared to other assessments that are often “just in a straight line” (C5), with clinicians specifically mentioning rapid acceleration, direction changes, turning or pivoting, and lateral footwork.

###### Cognitive Domain

Clinicians described cognitive elements of *R2Play* as uniquely sport-like compared to existing assessments. Specific cognitive skills they discussed included selected and divided attention, decision-making, multitasking, and quick reactions. Responding to spontaneous changes in the environment (ie, scramble condition) was highlighted as especially relevant to sport and informative for return-to-play assessment.

###### Perceptual Domain

Clinicians primarily discussed visual and auditory stimulation as perceptual elements of sport reflected in *R2Play*, specifically mentioning the background noise condition as an aspect that contributed to face validity.

### Missing Elements of Sport

Elements of sport that clinicians thought were missing from *R2Play* were sport-specific skills, navigating other competitors (ie, collision avoidance), communicating with teammates or coaches, visually tracking objects (ie, smooth pursuits), and hand-eye coordination.

### Suggestions to Improve Face Validity

Clinicians offered the following suggestions to enhance face validity of *R2Play*: incorporating sport-specific skills (eg, stick handling or dribbling between tablets) and surfaces (eg, testing on grass, turf, or court), having the athlete visually track and respond to an external stimulus while completing *R2Play* (eg, catching a ball), varying the heights of tablet targets to match sport-specific movement patterns, and displaying participants’ scores between levels to increase performance pressure.

### Qualitative Interview Findings (Youth)

#### Physical Domain

Youth discussed how dynamic movement during *R2Play* made it feel more like sport than other tests: “It’s moving around [as] opposed to walking and balancing on one foot, like I don’t normally balance on one foot when I’m playing a sport, I’m running around and I’m doing stuff and that’s kind of like me pressing the tablets” (Y8). They highlighted that agility skills used during *R2Play* reminded them of sport, including stopping and starting, pivoting, lunging, and quick direction changes. The cardiovascular demand of *R2Play*, involving short bursts of exertion followed by brief breaks, was also likened to sport.

#### Cognitive Domain

The “thinking” elements of *R2Play* made it feel more “realistic” for youth compared to other physical tests they had performed. Specific cognitive aspects that youth discussed were planning and monitoring plans, thinking and reacting quickly, and selected and divided attention. The scramble condition was highlighted as particularly relevant to sport-like thinking skills:

When the scramble happens, having to look around and reassess constantly where everything is as things change… having to move around and quickly shift what you're going to do and make a different plan without much warning… you gotta keep moving, but you also have to keep planning. That felt pretty sport-like.

[Y1]

#### Perceptual and Socioemotional Domains

Fewer youth discussed the perceptual and socioemotional aspects of *R2Play*, though some mentioned visual scanning, background noise, head movement, and competitiveness.

### Integrated Mixed Methods Face Validity Findings

**Figure 6.**
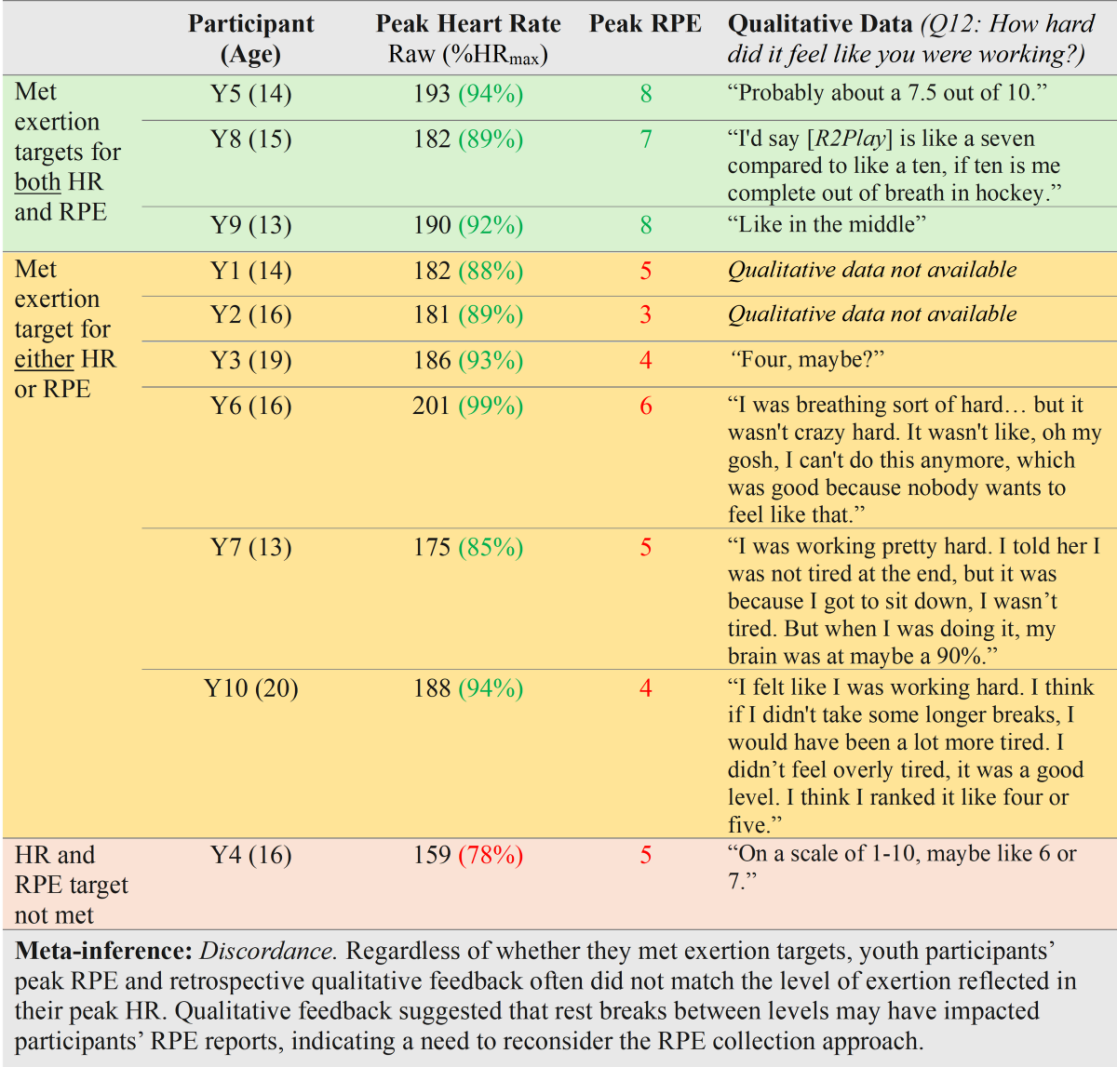
Cross-case comparison joint display of mixed methods integration for exertion data. Green text indicates the exertion target was met. Red text indicates the exertion target was not met. HR: heart rate; %HR_max_: percentage of age-predicted maximal heart rate; Q: question; RPE: rating of perceived exertion; Y: youth participant.

To address our mixing purposes of comparing, expanding, and enhancing*,* we constructed a cross-case comparison joint display [58,59] (Figure 6) linking youth participants’ physiological exertion (ie, HR), perceived exertion (RPE), and qualitative feedback. To better understand the relationship between these data sources and how well *R2Play* elicits a “sport-like” level of exertion, we stratified the joint display by whether participants met predefined exertion targets for HR and RPE (see Methods)*.*

## Discussion

### Overview

This study established initial feasibility and face validity of *R2Play*, a novel multidomain return-to-play assessment tool for concussion, at a single center through a mixed-methods approach involving a small sample of clinicians and youth with concussion history (Multimedia Appendix 6). Overall, most success criteria were met across all feasibility domains, with excellent usability (SUS=84.00±6.02), vigorous intensity exertion (peak HR=78%‐99% HR_max_), and no adverse events. Usability and technical issues were identified and addressed through iterative design changes, some of which are highlighted below. Clinicians and youth feedback indicated that *R2Play* reflects elements of sport across physical, cognitive, and perceptual domains, making it a potentially valuable tool for assessing readiness to return-to-play and informing rehabilitation treatment planning postconcussion.

### Feasibility

The mixed-methods approach and end user involvement in this study were particularly beneficial for understanding the usability and practicality of *R2Play*. Concordance across data sources indicated that *R2Play* is highly usable for both clinicians and youth (Table 6; meta-inference #1), which aligns with our proof-of-concept study findings [32]. This is critical given the importance of ease of use for successful adoption of rehabilitation technologies [61,62]. Importantly, we found that clinicians’ confidence in using *R2Play* was primarily limited by being unfamiliar with the assessment protocol and that support was needed to follow the protocol, deliver standardized instructions, and interpret and explain cost score results (meta-inference #2 and 4). These challenges have been addressed through upgrades to the clinician interface (map of assessment flow and standardized instruction scripts) and developing more extensive training resources. They also highlight an opportunity for participatory design and cocreation of knowledge mobilization materials to help clinicians communicate about *R2Play* in a manner that is meaningful for youth and families [63-65]. Consistent with broader usability literature [66,67], *R2Play* appeared to be used in an easier and faster manner with practice (meta-inference #3), reflecting calls to provide users with multiple opportunities to test a prototype [68]. Future work must consider changes in usability and *R2Play* results upon repeat administration (ie, test-retest reliability) due to familiarization with this novel approach. Overall, these findings reflect the value of mixed methods designs involving users in testing early prototypes of technology-based rehabilitation interventions and may be useful for other teams applying similar approaches [69].

The lack of adverse events and minimal symptom exacerbation during *R2Play* supports its safety*,* corroborated by youth and clinician feedback confirming their comfort with the protocol (meta-inference #6). Safety recommendations from clinicians, including preparticipation screening and monitoring tolerance, align with best practices for clinical exercise testing and postconcussion exertion assessment [50,51,70,71]. This feedback has informed an *R2Play* safety plan involving contraindication screening, standardized stopping criteria, and symptom management procedures. The absence of symptom provocation across most of this sample of recovered youth suggests that task requirements are appropriate and tolerable for youth beyond return-to-play clearance, providing a useful reference for future evaluation among youth with more recent concussion history. Importantly, 1 participant reported an increase in headache that may indicate incomplete physiological recovery [12,51], underscoring the potential for *R2Play* to detect unresolved changes that may be missed by traditional practices. Of note, this participant had a history of multiple concussions and was only 2 months postinjury at the time of testing. Further work is required to understand the safety of this approach among youth at the time of assessment for return-to-play clearance and establish the sensitivity of *R2Play* for detecting symptom exacerbation associated with ongoing concussion-related neurophysiological disturbances that may only manifest in complex testing environments involving integration across functional domains [72-74].

Qualitative feedback regarding acceptability and demand highlighted opportunities for *R2Play* to address gaps in the return-to-play process. Variability in operationalizing recovery and RTP noted by clinicians is well documented [11,75-77], reflecting fundamental challenges in this field [10,12]. Importantly, clinicians and youth expressed concerns about whether existing assessment and rehabilitation methods fully address the multidimensional aspects of return-to-play readiness, emphasizing the integration of physical and cognitive function as an element not adequately considered in practice, despite its ubiquity in sport [78] and growing attention in research [17,79]. The comprehensive nature of *R2Play* and its multidomain demands were thus appreciated by clinicians who viewed it as a potentially useful tool to assess tolerance to sport-like activity within a controlled setting before youth return to sport. As noted by clinicians, this assessment approach could involve monitoring physiological (HR) and symptom responses and capturing observations about movement quality and performance during prolonged dynamic exertion. Similarly, youth also saw *R2Play* as potentially useful to provide a clearer picture of readiness to return-to-play during advanced stages of recovery, which could help alleviate the anxiety and fear that are common when returning to sport [15,16]. In sum, clinicians and youth with concussion experience see value in novel multidomain assessments such as *R2Play* to help determine readiness for higher-risk sport activities (stages 4‐6 in the return-to-play protocol) [12] and to better capture the biopsychosocial factors that may contribute to reinjury [18,80] and limit psychological readiness to return-to-play [16,81]. These findings may be useful for other teams developing novel multidomain concussion assessments.

In this study and initial proof-of-concept work [32], some clinicians spontaneously identified the cost of *R2Play* as a potential implementation barrier. As cost estimates were not disclosed, these concerns may reflect preconceptions about technology, given the integration of multiple devices in *R2Play*. Nevertheless, cost is indeed an important factor that likely limits uptake of existing multidomain assessments, which often use sophisticated technologies to enhance ecological validity or enable scoring (eg, virtual reality and motion capture) [26]. Accessible pricing and equipment were therefore a priority in designing *R2Play* [28]. Equipment costs for the current *R2Play* prototype total approximately CAD $4250 (approximately US $3000) and are expected to decrease with further engineering refinement. This is markedly lower than many traditional neurocognitive testing batteries that face challenges in adoption due to high fees for software licensing, subscription, or interpretation [82]. Economic analyses are needed to establish the value of novel return-to-play tools such as *R2Play* by comparing the costs of assessment (in material and time) against potential savings in reducing reinjury and improving outcomes [83]. Commercialization models involving industry partnership could also help ensure sustainable pathways for widespread scalability of these tools.

### Physical Exertion and Face Validity

In this small sample of youth previously recovered from concussion, *R2Play* elicited vigorous intensity physical exertion (peak HR=78%‐99% HR_max_), with target exertion criteria met for 9/10 participants. This indicates that exertional demands are sufficient for assessing exercise tolerance, an important indicator of physiological recovery and readiness to return-to-play [51,84]. As expected, HR was highest in the exercise level, consistent with the physical demands. Interestingly, RPE was similar between the exercise and Stroop levels despite different physical demands. Possible explanations include (1) participants did not distinguish between physical exertion and cognitive effort in RPE ratings [85], (2) cognitive effort from the Stroop task caused higher perceptions of physical exertion [86], or (3) fatigue across the assessment led to higher-than-expected RPE during the final (Stroop) level [87]. Future work addressing these questions will obtain separate ratings of physical and cognitive effort (now built into the system) and explore perceptions of level demands using targeted interview questions.

Mixed method integration (Figure 6) revealed discordance wherein RPE and qualitative feedback often did not match participants’ peak HR. Here, we draw on mixed methods literature to address these discordant findings [88,89]. One approach is to seek explanations from existing research [90]. An exercise physiology lens may help understand the discrepancy between physiological (objective) and perceived (subjective) exertion responses. As *R2Play* involves short (~30‐60 s) intervals of high intensity exercise, it is possible that the brevity of these bouts and frequent breaks could limit perceptions of effort, despite substantially elevating HR. There is indeed evidence of RPE decoupling from HR during interval training, attributed to positive feelings of relief, achievement, and enjoyment [91,92]. This is particularly relevant because the youth described *R2Play* as enjoyable. Another strategy to manage discordance is comprehensive case analysis [88], a form of reconciliation (reanalysis) [89] that involves identifying patterns in individual case-level data. We speculate that sport history may influence participants’ effort perceptions such that those who are accustomed to explosive activities similar to *R2Play* (eg, hockey or soccer) may report lower effort perception. Given our limited sample size, initiation [89] of future work is warranted to explore this hypothesis by asking participants how the exertional demands of *R2Play* compare to their sports. Finally, an exclusion approach questions the validity of conflicting data [89]. In this case, temporal differences may have affected data quality. While HR was measured as participants actively completed *R2Play* levels, RPE and qualitative feedback were captured retrospectively while participants were at rest, during breaks, and upon assessment completion, respectively. Indeed, 2 participants mentioned that breaks affected their effort perception. In future work, care should be taken to ensure that RPE ratings pertain to active level completion.

### Limitations and Future Directions

This study lays the foundation for future work toward the goal of establishing *R2Play* as a clinically feasible, ecologically valid, and objective return-to-play assessment. Importantly, youth in this sample were previously cleared to RTP up to 5 years before enrollment, and clinicians did not administer *R2Play* in the context of real clinical decision-making for return-to-play clearance. While this work provided useful insights for the current stage of development, future validation studies should be conducted at the time of RTP clearance to determine the value of *R2Play* in supporting clinical decision-making (eg, increasing clinician confidence) and improving outcomes such as athletes’ psychological readiness to return-to-play and risk of reinjury. Additionally, youths’ concussion histories were established via self-report, without capturing how they received return-to-play clearance or what criteria were used to establish clearance. This information should be collected in future studies to contextualize participants’ *R2Play* results and symptom responses in relation to best practice guidelines and the established return-to-play strategy [12].

Future work is needed to validate the level structure, scoring, and ecological relevance of *R2Play*. This requires understanding the physiological, neurocognitive, and psychosocial task demands, which may be best accomplished through mixed-methods approaches that combine objective measurement with participant feedback [93]. Here, “sport-like” elements were primarily established through self-reported perceptions that may not translate to actual performance validity in real-world sport contexts. In addition to HR monitoring used here to capture physiological demands, objective measures of biomechanics (eg, accelerometry) and cognitive load (eg, electroencephalogram and functional near-infrared spectroscopy) could help assess whether movement and cognitive demands in *R2Play* reflect those in different sport environments. Validating a complex assessment such as *R2Play* is a complicated undertaking that is anticipated to span multiple years with ongoing iteration. This preliminary study was performed with a small convenience sample at a single site, supported by the original system design team, which limits generalizability. The primary author who conducted clinician training was also involved in data collection and interviews, which could influence participant responses (observer expectancy bias). Furthermore, we only considered a subset of relevant feasibility domains, some of which were only explored qualitatively. Future work will consider other aspects of feasibility (eg, implementation fidelity, adaptability, and integration) [39] across care settings, with quantitative progression criteria [59]. To address these questions, a larger multicenter pilot study is now underway evaluating the cross-site feasibility of *R2Play* and establishing its content validity by determining the physical, cognitive, and perceptual loading of the assessment leveling structure [94].

### Conclusions

Findings from this preliminary single-center study support the potential feasibility and face validity of *R2Play*, a new multidomain assessment tool for youth with concussion, demonstrating excellent usability, vigorous physical exertion demands, and promising feedback regarding its potential to fill gaps in the return-to-play process. Implementation considerations and mitigation strategies were identified related to time, cost, space, and equipment set-up. A multicenter study is underway to assess cross-site feasibility of *R2Play* and evaluate its content validity by establishing the physical, cognitive, and perceptual loading of assessment levels [94].

## Supplementary material

10.2196/78486 Multimedia Appendix 1 Joint display of integrated data collection.

10.2196/78486 Multimedia Appendix 2 *R2Play* cost scores.

10.2196/78486 Multimedia Appendix 3 Semistructured interview guides.

10.2196/78486 Multimedia Appendix 4 Observational analysis data extraction sheet.

10.2196/78486 Multimedia Appendix 5 PCSI and symptom check-in results. PCSI: postconcussion Symptom Inventory.

10.2196/78486 Multimedia Appendix 6 Visual abstract.

10.2196/78486 Checklist 1 GRAMMS checklist.

10.2196/78486 Checklist 2 Clinician training practice task checklist.

## Abbreviations

ABI: acquired brain injury
GRAMMS: Good Reporting of a Mixed Methods Study
HBKRH: Holland Bloorview Kids Rehabilitation Hospital
HR: heart rate
HR_max_: age-predicted maximal heart rate
PCSI: Post-Concussion Symptom Inventory
RPE: rating of perceived exertion
SUS: System Usability Scale
