# Supplementary material for: Evaluating R2Play, A Novel Multidomain Return-to-Play Assessment Tool for Concussion: Mixed Methods Feasibility and Face Validity Study
Source: JMIR Rehabil Assist Technol. 2025 Nov 25;12:e78486. doi: 10.2196/78486 (PMC12646560; doi:10.2196/78486)
Supplement: Multimedia Appendix 1 — Joint display of integrated data collection. [file rehab-v12-e78486-s001.docx]

**Objective 1: Feasibility**

| **Domain** | **Guiding questions** | **Data sources** | **Quantitative criteria** | **Qualitative interview questions** |
| --- | --- | --- | --- | --- |
| Acceptability | Do clinicians and youth judge *R2Play* to be suitable, appropriate, and satisfactory? | - Interviews | N/A | *Youth:*   - What do you think about our idea for *R2Play*? - What was your favorite part about it? What was your least favorite part about it?   *Clinician:*   - What do you think about our idea for *R2Play*? - What do you like about it? What do you not like? |
| Demand | Is *R2Play* likely to be used in the return-to-play process? | - Interviews | N/A | *Youth*   - Would you want to use *R2Play* as part of the RTP process?   *Clinician:*   - Do you think *R2Play* is an informative assessment? - Would you consider using *R2Play* in your practice? |
| Usability | Does the *R2Play* system allow clinicians and youth to complete the assessment effectively, efficiently, and to their satisfaction? | - SUS - Interviews - *R2Play* system log - Video recordings - Screen recordings - Field notes | - >80% on SUS - 90% of assessments completed without unrecoverable error | *Youth*   - Did you find it easy to use the *R2Play* tablet system?   *Clinician:*   - Did you find it easy to use the *R2Play* system overall? - Do you have any suggestions to improve the interface? |
| Reliability | Does the *R2Play* system perform appropriately to administer the assessment without error? | - *R2Play* system log - Video recordings - Screen recordings - Field notes | - 90% of assessments completed without major technical error | *Youth:*   - Did you notice any problems with the tablet buttons not working properly?   *Clinician:*   - Did you encounter any technical errors during the assessment? |
| Practicality | Can *R2Play* be administered in a timely and consistent manner? | - *R2Play* system log - Video recordings - Screen recordings - Interviews | - 90% of assessments completed within 15-30 minutes | *Youth:*   - What did you think about how long the assessment took?   *Clinician:*   - What did you think about how long the assessment took? |
| Safety | Can *R2Play* be administered safely to youth being cleared for RTP post-concussion? | - Video recordings - Field notes - Interviews | - No *R2Play*-related safety events requiring medical attention | *Youth*   - Did you have any worries as you completed the assessment?   *Clinician:*   - Do you think this assessment would be safe for athletes being evaluated for return to play clearance? - Is there anything you think should be done to ensure safety? |

**Objective 2: Face Validity**

| **Guiding question** | **Data sources** | **Quantitative criteria** | **Qualitative interview questions** |
| --- | --- | --- | --- |
| Does *R2Play* accurately simulate the demands of sport? | - Heart rate - Ratings of perceived exertion - Interviews | - Youth achieve 80% age- predicted HR_max_ | *Youth*   - Did the assessment remind you of playing sports? - Did it feel like you were working hard? For example, did your heart rate increase, did you have to think a lot? - Did you use any same skills that you use when playing sports?   *Clinician*:   - Do you think this is a fun assessment for youth athletes? - Do you think it emulates skills that are important in sport?   - Which skills?   - Are any critical aspects of sport missing? - Do you have any suggestions for how to make it more sport-like? |
