# Supplementary material for: Evaluating R2Play, A Novel Multidomain Return-to-Play Assessment Tool for Concussion: Mixed Methods Feasibility and Face Validity Study
Source: JMIR Rehabil Assist Technol. 2025 Nov 25;12:e78486. doi: 10.2196/78486 (PMC12646560; doi:10.2196/78486)
Supplement: Multimedia Appendix 2 — R2Play cost scores. [file rehab-v12-e78486-s002.docx]

| Cost score | Increasing load | Comparison | Calculation** |
| --- | --- | --- | --- |
| Exertion cost | Physical | Number-Letter level (standard condition) Vs. Exercise level (standard condition)* | [Completion time (Exercise level) – Completion time (Number-Letter Level)] / Completion time (Number-Letter level) x 100% |
| Cognitive cost | Cognitive | Number-Letter level (standard condition) Vs. Stroop level (standard condition)* | [Completion time (Stroop level) – Completion time (Number-Letter level)] / Completion time (Number-Letter level) x 100% |
| Auditory interference cost | Perceptual | Average change between standard condition Vs. auditory interference condition across all levels | Average of [(Completion time (auditory condition rep) – Completion time (standard condition rep)] / Completion time (standard condition rep) for all levels x 100% |
| Scramble cost | Multi-domain switching | Average change between standard condition Vs. scramble condition across all levels | Average of [(Completion time (scramble condition rep) – Completion time (standard condition rep)] / Completion time (standard condition rep) for all levels x 100% |
| Fatigue cost (mid) | Fatigue | Fatigue check (pre) Vs. Fatigue check (mid)* | [Completion time (fatigue check mid) – Completion time (fatigue check pre)] / Completion time (fatigue check pre) x 100% |
| Fatigue cost (post) | Fatigue | Fatigue check (pre) Vs. Fatigue check (post)* | [Completion time (fatigue check post) – Completion time (fatigue check pre)] / Completion time (fatigue check pre) x 100% |

Avg: average; Reps: Repetitions. *Comparison uses best performance of two repetitions for standard condition and motor task. **Calculations also computed for heart rate and errors.
