# Supplementary material for: Evaluating R2Play, A Novel Multidomain Return-to-Play Assessment Tool for Concussion: Mixed Methods Feasibility and Face Validity Study
Source: JMIR Rehabil Assist Technol. 2025 Nov 25;12:e78486. doi: 10.2196/78486 (PMC12646560; doi:10.2196/78486)
Supplement: Multimedia Appendix 3 — Semistructured interview guides. [file rehab-v12-e78486-s003.docx]

**Youth Interview Guide**

The purpose of the interview is to see what you thought about our assessment. The questions are open, and you can bring up anything you think is important. You are the expert here and we are looking to learn from you, so please give us your honest opinions and not just what you think we want to hear. You do not have to answer any question that makes you feel uncomfortable, and you can stop the interview at any time. The interview will be audio recorded so that the research team can keep a record of the interview. Your responses will be confidential within the research team and will not be shared with your parents, coaches, or teachers. We won’t use your name in any findings we publish. We might use some quotes from the interviews, but we will not say your name. This will let our team review it and analyze the responses along with other participants’ responses to help with our research study. Do you have any questions for me?

| **Focus area** | **Example comments and questions** |
| --- | --- |
| Introduction | 1. What are your favorite sports that you like to play? 2. Can you tell me a little bit about what it’s been like returning to sport after your concussion?    1. What were some of the different concussion tests that you did as part of the return to sport process?    2. How ready did you feel to return to playing sport after your concussion? |
| Acceptability | 1. Tell me what you think about *R2Play* in general. 2. How does *R2Play* compare to other tests you have done in the return to play process after concussion? 3. What was your favorite part of *R2Play*? 4. What was your least favorite part? 5. How fun was *R2Play*? |
| Usability and Reliability | 1. Tell me about your experience using the tablet buttons in *R2Play* 2. How easy did you find it to use the tablet system? 3. Did you notice any problems with the tablet buttons not working properly? |
| Face Validity | 1. Did *R2Play* give you the feeling like you were playing a sport? 2. How hard did it feel like you were working?    1. Were you breathing hard? Did you have to think a lot? 3. In what ways do you think *R2Play* mimics the skills that you use when playing sports? |
| Demand | 1. How do you think *R2Play* could be used during the recovery and return to sport process after concussion? 2. Could you describe how R*2Play* might have helped you feel more ready to return to play after your concussion? |
| Practicality | 1. What do you think about how long the assessment took?    1. Did it feel too long or too short? 2. How easy was it for you to learn the rules for *R2Play*?    1. Thinking about the instructions that were provided to you during the test, what are some things that could have been explained more clearly to you? |
| Safety | 1. Can you describe how you felt during the *R2Play* test?    1. Did you have any worries?    2. Did you feel safe?    3. Were you nervous? |
| Additional Thoughts | 1. Is there anything else you would like to tell us about your experience using *R2Play* today? |

**Clinician Post-Assessment Interview Guide**

The purpose of this interview is a quick reflection on your experience with the *R2Play* assessment. We will be following up again with a phone call after your second session, so we can get a more in-depth understanding of what you think about the assessment. For now, we just want your first impressions! The interview will be audio recorded. You do not have to answer any question you feel uncomfortable with, and you can stop the interview at any time. Your responses will be confidential and anonymized in any findings we publish. Quotes from the interviews may be used, but it will not be possible to identify their source. The interview will be audio recorded so that the research team can keep a record of the interview. This will let the team review it and analyze the responses along with other participants’ responses to help with our research study. Do you have any questions for me?

| **Focus area** | **Example comments and questions** |
| --- | --- |
| Result interpretation | 1. Do you think the assessment would be informative in RTP decision making? 2. If this individual had been recently concussed how would you use these results to inform an RTP clearance decision? 3. Did you think the results were useful? 4. Would you like to have seen any other results? 5. How does *R2Play* compare to other assessments you have used in the return to play process? |
| System Refinement | 1. Do you think the levels were in a good order or sequence? If you could choose, would you have done them in a different way? |
| Training | 1. What do you think about the training you were given to learn the *R2Play* assessment?    1. How well do you think it prepared you to deliver the assessment? 2. How confident did you feel delivering the assessment?    1. What could we have done to improve your confidence? 3. Do you have any suggestions for how we can improve the training? |

**Clinician Follow-Up Interview Guide**

The purpose of this interview is to reflect on your experience with the *R2Play* assessment. The questions will be open-ended to allow you the chance to raise the issues that you feel are important. You are the expert here and we are looking to learn from you, so please give us your honest opinions and not just what you think we want to hear. You do not have to answer any question you feel uncomfortable with, and you can stop the interview at any time. The interview will be audio recorded so that the research team can keep a record of the interview. This will let the team review it and analyze the responses along with other participants’ responses to help with our research study. Your responses will be confidential and anonymized in any findings we publish. Quotes from the interviews may be used, but it will not be possible to identify their source. Do you have any questions for me?

| **Focus area** | **Example comments and questions** |
| --- | --- |
| Acceptability | 1. What do you think about our idea for the *R2Play* assessment?    1. What did you like about it?    2. What didn’t you like about it? |
| Usability and Reliability | 1. How would you describe your overall experience using the *R2Play* system?    1. Can you describe the aspects you found were easy to use?    2. Which features were more difficult for you? 2. Do you have any suggestions to improve the clinician interface? 3. Can you describe any technical errors you encountered during the assessment? |
| Demand | 1. How informative do you think the *R2Play* assessment is for return to play decision making?    1. How could we make it more informative? 2. How useful do you think the scoring measures and results are?    1. What other results might you have wanted to see? 3. How do you think *R2Play* compares to other assessments used in the return to play process? 4. How might you consider using *R2Play* in your practice? |
| Face validity | 1. How fun do you think the assessment is for youth athletes? 2. How well do you think it emulates skills that are important in sport?    1. Which skills do you think are reflected well in *R2Play*?    2. Are any critical aspects of sport missing? 3. What suggestions do you have for how to make it more sport-like? |
| Safety | 1. How safe do you think this assessment would be for athletes being evaluated for return to play clearance?    1. What do you think would need to be done to ensure safety? |
| Implementation  and Practicality | 1. What do you think about the length of the assessment?    1. Was it too long or too short?    2. How long do you think the assessment should be?    3. Were there any parts that you felt were not useful and could be removed? 2. What do you think would need to be done to support the implementation of *R2Play* in practice?    1. What do you think would help facilitate implementation?    2. Do you foresee any other barriers to implementing *R2Play*?    3. What could be done to overcome these barriers? |
| Expansion | 1. Rather than only a final return to play assessment, how do you think *R2Play* could be used as more of a proactive rehabilitation tool to guide personalized therapy planning? |
| Adaptation | 1. How well do you think *R2Play* could be adapted for different populations? For example, para-athletes, younger kids, non-athletes, or adult athletes?    1. How would you suggest it be adapted for them? |
| Training | 1. What do you think about the training you were given to learn the *R2Play* assessment?    1. How well do you think it prepared you to deliver the assessment? 2. What do you think about the content of the training session?    1. Is there any other information you would have wanted to receive during training? 3. What do you think about the format of the training session?    1. Were you able to learn the assessment effectively through the virtual training session?    2. Would you have preferred a different format of training? 4. How do you think we could improve the training? 5. Was there anything else beyond the formal training that helped you learn how to deliver the assessment? 6. If you were to implement this assessment in your practice, is there any further training or support that you think you would need? |
| Additional Thoughts | 1. Is there anything else you would like to tell us about your experience using *R2Play* today? 2. Do you have any other suggestions to improve *R2Play*? |
