# Supplementary material for: Evaluating R2Play, A Novel Multidomain Return-to-Play Assessment Tool for Concussion: Mixed Methods Feasibility and Face Validity Study
Source: JMIR Rehabil Assist Technol. 2025 Nov 25;12:e78486. doi: 10.2196/78486 (PMC12646560; doi:10.2196/78486)
Supplement: Multimedia Appendix 4 — Observational analysis data extraction sheet. [file rehab-v12-e78486-s004.docx]

*R2Play* sessions are video recorded to enable review of system performance all interactions between the clinician, youth, and research team. Screen recordings of the clinician laptop are also taken to capture clinician interactions with the *R2Play* interface. For each *R2Play* session, screen and video recordings will be reviewed and cross-referenced to identify any technical errors or usability issues.

**Event definitions**

Technical errors refer to instances in which the system malfunctions or does not perform appropriately as expected (e.g., unresponsive tablet, incorrect content displayed on tablet, wrong screen shown within clinician interface, buttons within interface not functioning properly).

Usability issues refer to aspects of the system or a user’s (clinician or youth) interaction with the system that make it difficult for them to independently complete an assessment task effectively and efficiently (e.g., pressing wrong button to achieve desired goal or not understanding level instructions).

**Event severity**

The severity of technical errors and usability issues should be classified using the following options.

- **Recoverable**: System or interface enters a state that the users did not intend but they can quickly recover and continue the assessment without intervention from the research team.
- **Unrecoverable**: System or interface enters a state that the users did not intend, and they cannot recover without intervention from the research team. The research team resolves the issue quickly without significantly interrupting the assessment session.
- **Catastrophic**: Assessment session is terminated and must be entirely re-started or significantly interrupted in a way that requires substantial intervention from the research team and causes a delay in continuing the assessment.

The following table provides examples of events that may be identified via video and screen recordings.

|  | **Technical errors** | **Usability issues** |
| --- | --- | --- |
| Screen recording | - Interface responds slowly or does not respond to input - Wrong screen shows up within the clinician interface - Buttons do not function properly within clinician interface | - Accidentally presses the wrong button within interface - Navigates to the wrong screen within the interface - Difficulty making selections within the interface |
| Video recording | - Tablet is unresponsive or does not register selection from youth - System lags or is slow to respond - Incorrect content is displayed on a tablet - Audio cue plays at the wrong time | - Youth does not understand instructions and requires further explanation from clinician or researcher *during* a repetition. Direction provided during training or between reps does not count as a usability issue. - Tablet falls off stand or needs adjustment - Clinician needs help from research team |

**Reviewer: ______________________**

**Session number**: ____________________

**Technical and usability events**

| **Time stamp** | **Type of event** | **Severity** | **Description of event** |
| --- | --- | --- | --- |
|  | Technical error  Usability issue | Recoverable  Unrecoverable  Catastrophic |  |
|  | Technical error  Usability issue | Recoverable  Unrecoverable  Catastrophic |  |
|  | Technical error  Usability issue | Recoverable  Unrecoverable  Catastrophic |  |
|  | Technical error  Usability issue | Recoverable  Unrecoverable  Catastrophic |  |
