# Supplementary material for: Evaluating R2Play, A Novel Multidomain Return-to-Play Assessment Tool for Concussion: Mixed Methods Feasibility and Face Validity Study
Source: JMIR Rehabil Assist Technol. 2025 Nov 25;12:e78486. doi: 10.2196/78486 (PMC12646560; doi:10.2196/78486)
Supplement: Multimedia Appendix 5 — PCSI and symptom check-in results. PCSI: postconcussion Symptom Inventory. [file rehab-v12-e78486-s005.docx]

| **Participant** | **PCSI Score** | | | **Symptom Check-In Score*** | | |
| --- | --- | --- | --- | --- | --- | --- |
|  | **Pre-Assessment** | **Post-Assessment** | **Change** | **Number Letter Level** | **Exercise Level** | **Stroop Level** |
| Y1 | 12 | 12 | 0 | 1 | 2 | 2 |
| Y2 | 5 | 5 | 0 | 1 | 1 | 1 |
| Y3 | 10 | 3 | -7 | 0 | 0 | 0 |
| Y4 | 15 | 29 | +14 | 1 | 1 | 1 |
| Y5 | 11 | 6 | -5 | 0 | 0 | 0 |
| Y6 | 17 | 14 | -3 | 0 | 0 | 0 |
| Y7 | 5 | 1 | -4 | 0 | 0 | 0 |
| Y8 | 5 | 6 | +1 | 0 | 0 | 0 |
| Y9 | 14 | 6 | -8 | 0 | 0 | 0 |
| Y10 | 10 | 5 | -5 | 0 | 0 | 0 |
| Median (IQR) | 10.5 (5-14) | 6 (5-12) | -3.5 (-5-0) | 0 (0-1) | 0 (0-1) | 0 (0-1) |

*0 = No symptoms, 1 = Some symptoms but did not get worse, 2 = Symptoms got worse, 3 = Symptoms so much worse I had to stop.
