# Supplementary figures and images for: Evaluating R2Play, A Novel Multidomain Return-to-Play Assessment Tool for Concussion: Mixed Methods Feasibility and Face Validity Study
Source: JMIR Rehabil Assist Technol. 2025 Nov 25;12:e78486. doi: 10.2196/78486 (PMC12646560; doi:10.2196/78486)

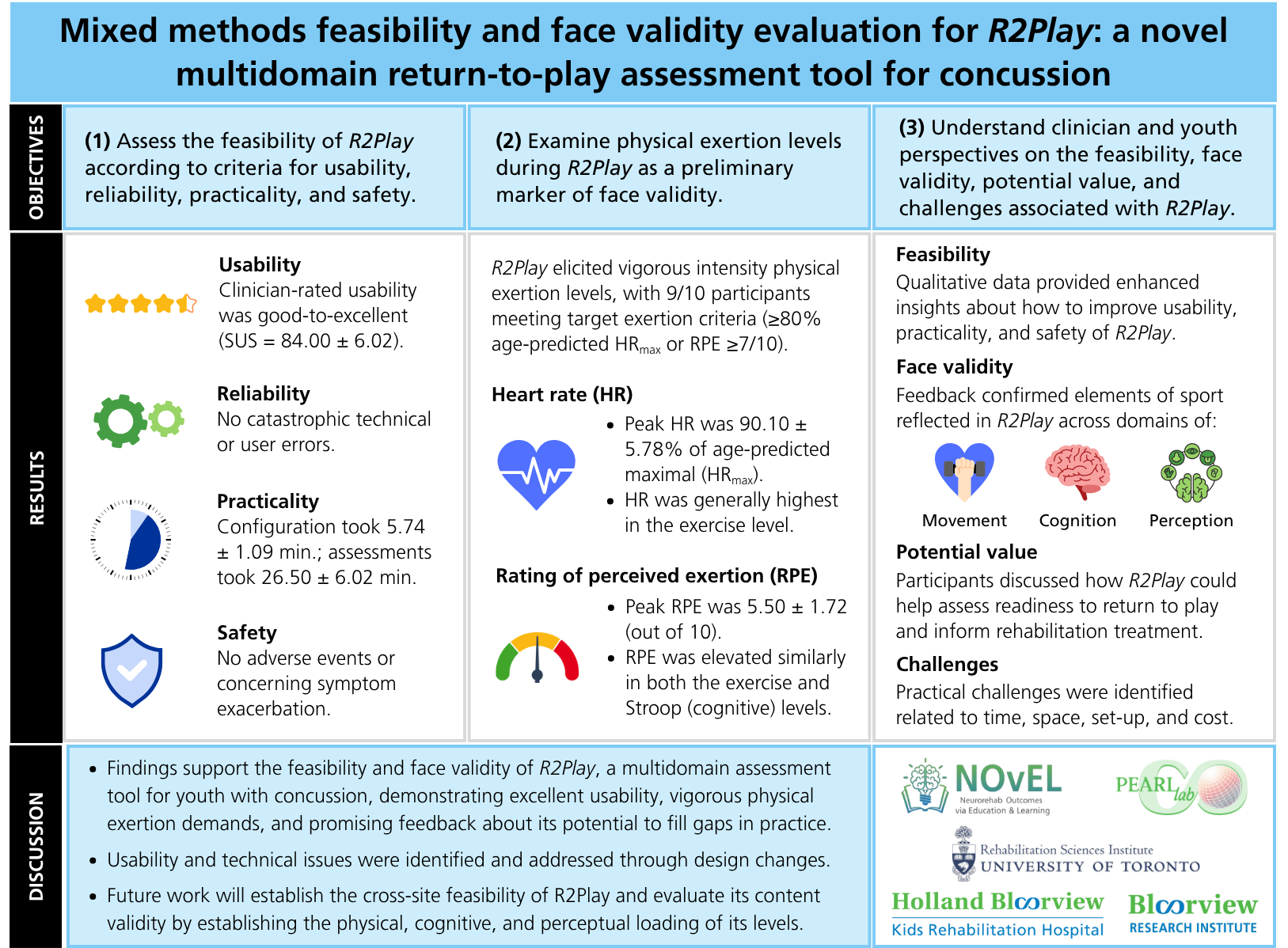

Supplement: Multimedia Appendix 6 — Visual abstract. [file rehab-v12-e78486-s006.png]
