# Supplementary material for: Evaluating R2Play, A Novel Multidomain Return-to-Play Assessment Tool for Concussion: Mixed Methods Feasibility and Face Validity Study
Source: JMIR Rehabil Assist Technol. 2025 Nov 25;12:e78486. doi: 10.2196/78486 (PMC12646560; doi:10.2196/78486)
Supplement: Checklist 1 — GRAMMS checklist. [file rehab-v12-e78486-s007.docx]

| **Recommended Element** | **Location in Manuscript** (Section – Sub-Section: Page) |
| --- | --- |
| Describe the justification for using a mixed methods approach to the research question(s) | Methods – Design: Pg. 4 |
| Describe the design in terms of the purpose, priority and sequence of methods | Methods – Design: Pg. 4 |
| Describe each method in terms of sampling, data collection and analysis | Methods – Participants, Recruitment, Protocol, Data Collection Measures, Analysis: Pg. 5-9 |
| Describe where integration has occurred, how it has occurred and who has participated in it | Methods – Mixed methods integration: Pg. 9  Results - Integrated Mixed Methods Feasibility Findings: Pg. 16-17.  Results – Integrated Mixed methods face validity findings: Pg. 20-21. |
| Describe any limitation of one method associated with the presence of the other method | Discussion – Limitations and Future Directions: Pg. 23. |
| Describe any insights gained from mixing or integrating methods | Results - Integrated Mixed Methods Feasibility Findings: Pg. 16-17.  Results – Integrated Mixed methods face validity findings: Pg. 20-21.  Discussion – Feasibility: Pg. 21-22.  Discussion – Face validity: Pg. 22-23. |

**Reference**:

40. O'Cathain A, Murphy E, Nicholl J. The quality of mixed methods studies in health services research. J Health Serv Res Policy. 2008;13: 92-98.
