# Supplementary material for: Evaluating R2Play, A Novel Multidomain Return-to-Play Assessment Tool for Concussion: Mixed Methods Feasibility and Face Validity Study
Source: JMIR Rehabil Assist Technol. 2025 Nov 25;12:e78486. doi: 10.2196/78486 (PMC12646560; doi:10.2196/78486)
Supplement: Checklist 2 — Clinician training practice task checklist. [file rehab-v12-e78486-s008.docx]

| **Task** | **Completed with ease (2 points)** | **Completed with help (1 point)** | **Did not complete (0 points)** |
| --- | --- | --- | --- |
| *Setting up assessment* | | | |
| 1. Access the client library and create an athlete profile for a new client. |  |  |  |
| 1. Add a new assessment room with specified dimensions. |  |  |  |
| *Administering assessment* | | | |
| 1. Begin a new assessment session with a specified customized trail length. |  |  |  |
| 1. Administer baseline assessment of resting heart rate and concussion symptoms. |  |  |  |
| 1. Train a new client on a given level of the assessment. |  |  |  |
| 1. Document a clinical observation at a specified point during an assessment. |  |  |  |
| 1. Conduct a check-in assessment of a client’s RPE and concussion symptoms. |  |  |  |
| *Reviewing assessment results* | | | |
| 1. Report the peak and average heart rate that a client achieved in an assessment. |  |  |  |
| 1. Describe the change in a client’s symptoms during an assessment. |  |  |  |
| 1. Report and explain a specified cost score from an assessment to a new client. |  |  |  |
| **Total score** | **/** 20 *(sum score from each task)* | | |
